# THADA inhibition in mice protects against type 2 diabetes mellitus by improving pancreatic β-cell function and preserving β-cell mass

**DOI:** 10.1038/s41467-023-36680-0

**Published:** 2023-02-23

**Authors:** Yuqing Zhang, Shan Han, Congcong Liu, Yuanwen Zheng, Hao Li, Fei Gao, Yuehong Bian, Xin Liu, Hongbin Liu, Shourui Hu, Yuxuan Li, Zi-Jiang Chen, Shigang Zhao, Han Zhao

**Affiliations:** 1 Center for Reproductive Medicine, Shandong University, 250012 Jinan, Shandong China; 2 Key Laboratory of Reproductive Endocrinology of Ministry of Education, Shandong University, 250012 Jinan, Shandong China; 3 Shandong Key Laboratory of Reproductive Medicine, Shandong Provincial Hospital Affiliated to Shandong First Medical University, 250012 Jinan, Shandong China; 4 Department of Hepatobiliary Surgery, Shandong Provincial Hospital Affiliated to Shandong First Medical University, 250021 Jinan, Shandong China; 5 Shandong Provincial Qianfoshan Hospital, Shandong University, 250014 Jinan, Shandong China; 6 State Key Laboratory of Reproductive Biology, Institute of Zoology, Chinese Academy of Science, 100101 Beijing, China; 7 Shanghai Key Laboratory for Assisted Reproduction and Reproductive Genetics, 200135 Shanghai, China; 8 Research Unit of Gametogenesis and Health of ART-Offspring, Chinese Academy of Medical Sciences (No. 2021RU001), Shandong 250012 Jinan, China

**Keywords:** Type 2 diabetes, Mechanisms of disease, Apoptosis

## Abstract

Impaired insulin secretion is a hallmark in type 2 diabetes mellitus (T2DM). *THADA* has been identified as a candidate gene for T2DM, but its role in glucose homeostasis remains elusive. Here we report that THADA is strongly activated in human and mouse islets of T2DM. Both global and β-cell-specific *Thada*-knockout mice exhibit improved glycemic control owing to enhanced β-cell function and decreased β-cell apoptosis. THADA reduces endoplasmic reticulum (ER) Ca^2+^ stores in β-cells by inhibiting Ca^2+^ re-uptake via SERCA2 and inducing Ca^2+^ leakage through RyR2. Upon persistent ER stress, THADA interacts with and activates the pro-apoptotic complex comprising DR5, FADD and caspase-8, thus aggravating ER stress-induced apoptosis. Importantly, THADA deficiency protects mice from high-fat high-sucrose diet- and streptozotocin-induced hyperglycemia by restoring insulin secretion and preserving β-cell mass. Moreover, treatment with alnustone inhibits THADA’s function, resulting in ameliorated hyperglycemia in obese mice. Collectively, our results support pursuit of THADA as a potential target for developing T2DM therapies.

## Abstract (web summary)

THADA has been identified as a type 2 diabetes-associated gene whose function is not fully understood. Here the authors report that THADA deficiency protects mice from hyperglycemia and glucose intolerance by promoting insulin secretion and inhibiting β-cell apoptosis, suggesting THADA could be explored as a potential therapeutic target for diabetes.

## Introduction

Type 2 diabetes mellitus (T2DM) is currently a fast-growing health problem affecting nearly half a billion people worldwide. It is characterized by hyperglycemia due to insufficient insulin secretion by pancreatic β-cells in response to overnutrition or insulin resistance^1^. T2DM were driven by the interplay of genetic and environmental risk factors, with pancreatic islet being considered as the key tissue in mediating its pathogenesis^2^. Impaired β-cell function and inadequate β-cell mass underpins the main cause of this disease^3,4^. Therefore, understanding the molecular mechanism that drives pathological β-cell defect is urgently needed to develop strategies for the prevention and treatment of T2DM.

The *Thyroid Adenoma Associated* (*THADA*), target gene of chromosomal aberration in thyroid adenomas^5^, has been identified as a T2DM-associated gene by several genome-wide association studies (GWAS)^6–8^. *THADA* gene variants showed excess maternal transmission to offspring with T2DM, indicative of a disease-specific effect^9^. *THADA* was detected to be associated with lower pancreatic β-cell response^10^, but not with insulin sensitivity^11^. In a Chinese population, it was correlated with 2-h insulin levels during oral glucose tolerance tests^12^. Moreover, Single nucleotide polymorphisms (SNPs) in *THADA* were associated with polycystic ovary syndrome (PCOS)^13^ as well as cold adaption during human evolution^14^. All these findings connected THADA to energy homeostasis.

Studies of the physiological functions of THADA are limited. Moraru et al. (2017) revealed that *THADA* knockout in *Drosophila* led to obesity due to hyperphagia, reduced thermogenesis and increased lipid storage^15^. They identified that THADA uncouples ATP hydrolysis from Ca^2+^ transport into the endoplasmic reticulum (ER), which results in thermogenesis due to dissipation of energy in the form of heat^15^. Another study reported that depletion of *Arabidopsis* THADA homolog caused hypersensitivity to cold^16^. THADA protein was predicted to have the ARM repeat structure which indicates the protein binding activity^17^, and encode a death receptor-interacting protein (Puduvalli VK and Ridgeway L, GenBank reference note). Nonetheless, the metabolic impacts of THADA in mammals have not been reported to date. In vivo characterizing THADA’s function in mammals could help unveil its role in the pathogenesis of metabolic diseases and provide therapeutic hints.

In the present study, we generated global and β-cell-specific *Thada*-knockout mice and investigated the effects of THADA deficiency on glucose homeostasis under physiological and diabetic conditions. We report that THADA impairs insulin secretion and β-cell survival by decreasing ER Ca^2+^ stores and aggravating ER stress-induced apoptosis. Moreover, THADA deficiency protected mice from high-fat high-sucrose (HFHS) diet- and streptozotocin (STZ)-induced T2DM. Our findings highlight THADA as a determinant for β-cell secretory function and β-cell mass, thus providing a potential therapeutic target for T2DM.

## Results

### THADA is activated in T2DM islets and after glucolipotoxicity

**Fig. 1:**
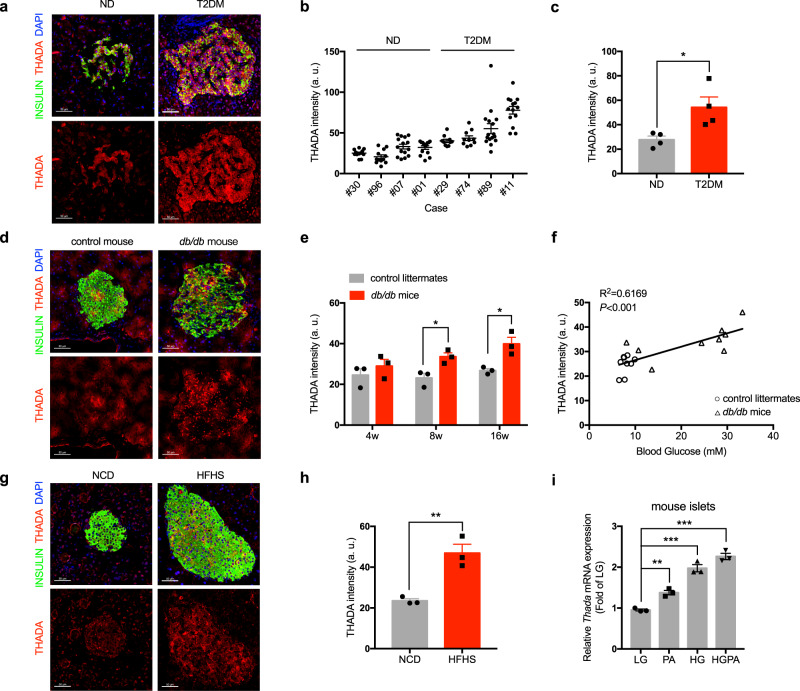
THADA is activated in islets of T2DM patients and mice. **a** Representative pancreatic sections from non-diabetes (ND) and type 2 diabetes mellitus (T2DM) participants stained for insulin (green), THADA (red), and DAPI (blue). Scale bars, 50 μm. **b** THADA intensity of each islet in ND and T2DM participants (*n* = 10 for #29 and #74, *n* = 12 for #96, *n* = 13 for #30, *n* = 14 for #01 and #11, *n* = 15 for #07, *n* = 16 for #89). **c** Quantification of mean THADA intensities in ND and T2DM individuals (*n* = 4). **d** Representative pancreatic sections from 16-week-old *db/db* mice and their wild-type control littermates stained for insulin (green), THADA (red), and DAPI (blue). Scale bars, 50 μm. **e** Quantification of THADA intensities in control littermates and *db/db* mice at 4, 8, and 16 weeks of age (*n* = 3). **f** Linear regression analysis of islet THADA intensities and blood glucose levels in control littermates (circles) and *db/db* mice (triangles). The *R*^2^ and *P* value were shown in panel. **g** Representative pancreatic sections from mice feeding HFHS diet for 12 weeks or normal chow diet (NCD) staining for insulin (green), THADA (red), and DAPI (blue). Scale bars, 50 μm. **h** Quantification of mean THADA intensities in NCD- and HFHS diet-fed mice (*n* = 3). **i** *Thada* mRNA expression in mouse islets incubated with 3.3 mM glucose (LG), 0.5 mM palmitate (PA), 16.7 mM glucose (HG), or 16.7 mM glucose combined with 0.5 mM palmitate (HGPA) for 48 h (*n* = 3 biologically independent experiments). Data are presented as mean ± SEM. *P < 0.05, **P < 0.01, ***P < 0.001; significance is assessed by two-tailed unpaired Student’s *t* test (**c**, **e**) or one-way ANOVA followed by Dunnett’s multiple comparison test (**i**). Source data are provided as a Source Data file.

We observed *Thada* is highly expressed in mouse islets (Fig. S1a), which prompted us to evaluate its expression in pancreatic sections from T2DM patients and non-diabetic (ND) control participants. Detailed clinical profiles for these participants are provided in Supplementary Data 1. The islet from T2DM patients displayed strong expression of THADA, specifically in the cytosol of insulin-positive β-cells, not in exocrine cells (Fig. 1a). Further, the THADA immunofluorescence intensity was substantially increased in islets from T2DM patients compared to islets from the ND participants (Fig. 1b, c).

We next assessed whether THADA is involved in the progression of T2DM by examining *db/db* mice—a spontaneous T2DM mouse model—at different ages (Fig. S1b). We found THADA was expressed mainly in islet β-cells, not in α-cells (Fig. S1c). Its expression level in islet was indistinguishable between 4-week-old prediabetic *db/db* mice and wild-type littermate controls, but gradually increased when the *db/db* mice progressed into T2DM at 8 and 16 weeks (Fig. 1d, e), indicating THADA is activated during diabetes progression. Moreover, we detected a strong positive correlation between THADA expression levels and fed blood glucose levels (Fig. 1f). We observed a similar pattern of increased THADA expression in another T2DM mouse model induced by a HFHS diet (Fig. 1g, h).

Chronic hyperglycemia and hyperlipidemia, collectively referred to as “glucolipotoxicity”, has been proposed as a driver of T2DM^18^. To explore potential relationships between THADA expression and glucolipotoxicity, we simulated this diabetogenic milieu in mouse islets in vitro. Chronic exposure to high concentrations of glucose and palmitate significantly induced *Thada* expression in mouse islets (Fig. 1i and S1d). A high concentration of glucose or palmitate alone could also induce *Thada* expression in mouse islets or cultured β-cells (Fig. 1i, S1e and S1f). We obtained similar results when these β-cells were exposed to a 2:1 mixture of oleate/palmitate (Fig. S1g). These data suggest that THADA is activated in diabetogenic conditions.

### *Thada* knockout improves glucose homeostasis without affecting insulin sensitivity in mice

**Fig. 2:**
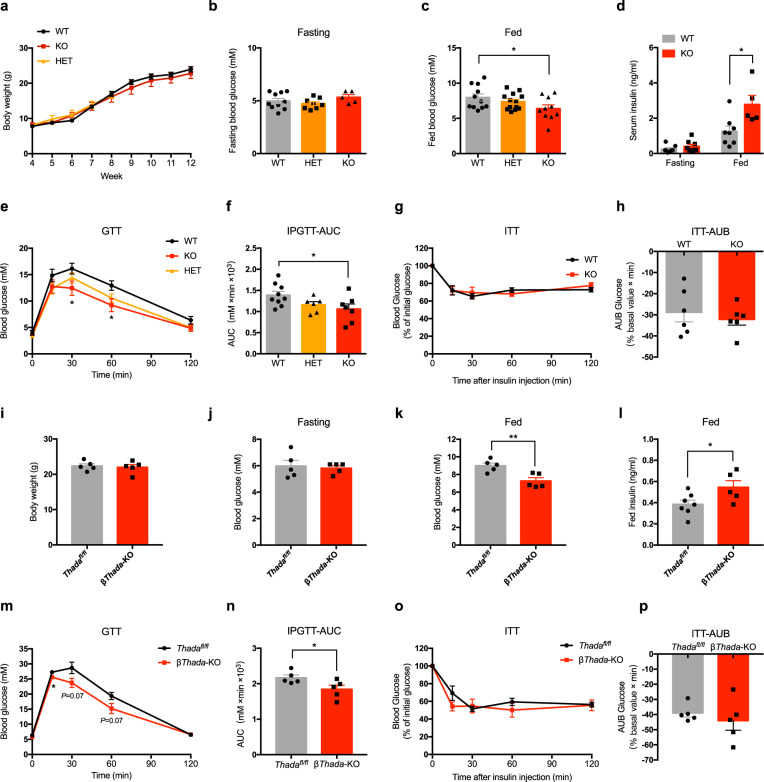
*Thada* knockout improves glucose homeostasis without affecting insulin sensitivity in mice. **a** Body weights of wild-type (WT, *n* = 13), heterozygous (HET, *n* = 13), and *Thada*-knockout (KO, *n* = 11) mice at the indicated weeks of age. **b** Fasting blood glucose levels of WT (*n* = 10), HET (*n* = 8) and *Thada*-KO mice (*n* = 5) that were fasted overnight. **c** Random-fed blood glucose levels of WT (*n* = 11), HET (*n* = 13), and *Thada*-KO mice (*n* = 10). **d** Fasting (*n* = 8) and fed (*n* = 8 for WT and *n* = 5 for KO) serum insulin levels of WT and *Thada*-KO mice. **e**, **f** IPGTT and the related area under the curve (AUC) of WT (*n* = 9), HET (*n* = 6) and *Thada*-KO mice (*n* = 7). **g**, **h** ITT and the related area under baseline (AUB) of WT and *Thada*-KO mice (*n* = 6). (**i**) Body weights of control (*Thada*^*fl/fl*^) and β-cell-specific *Thada*-knockout (β*Thada*-KO) mice (*n* = 5). **j**, **k** 16-h fasting blood glucose (**j**) and random-fed blood glucose (**k**) levels of *Thada*^*fl/fl*^ and β*Thada*-KO mice (*n* = 5). **l** Fed serum insulin levels of *Thada*^*fl/fl*^ (*n* = 7) and β*Thada*-KO (*n* = 5) mice. **m**, **n** IPGTT and the related AUC of *Thada*^*fl/fl*^ and β*Thada*-KO mice (*n* = 5). **o**, **p** ITT and the related AUB of *Thada*^*fl/fl*^ and β*Thada*-KO male mice (*n* = 5). Experiments were performed on male mice at 8–10 weeks of age and female mice data were provided in Fig. S2. Data are presented as mean ± SEM. *P < 0.05, **P < 0.01; significance is assessed by two-tailed unpaired Student’s *t* test. Source data are provided as a Source Data file.

We next generated mice with whole-body *Thada* knockout by CRISPR/Cas9 (Fig. S2a) and confirmed successful THADA deletion (Fig. S2b–d). There were no significant differences in the body weights of wild-type (WT) and *Thada*-knockout (*Thada*-KO) mice (Fig. 2a). Nor did *Thada*-KO cause any differences in the weights of liver, epididymal, or inguinal white adipose tissues, as well as food intake of mice (Fig. S2e, f). However, although *Thada*-KO mice exhibited similar fasting glucose and insulin levels as their WT littermates (Fig. 2b, d), they showed significantly decreased random-fed blood glucose levels (Fig. 2c). And this was accompanied with significantly increased serum insulin levels (Fig. 2d). We next measured blood glucose levels after intraperitoneal glucose injection. *Thada*-KO mice showed significantly lower blood glucose compared with WT controls during the intraperitoneal glucose tolerance test (IPGTT) (Fig. 2e, f). An intraperitoneal insulin tolerance test (ITT) showed no significant difference in insulin sensitivity between WT and *Thada*-KO mice (Fig. 2g, h). Female *Thada*-KO mice displayed similar phenotypes as male mice (Fig. S2g–n).

We further constructed β-cell-specific *Thada*-knockout (β*Thada*-KO) mice (Fig. S2o) and found that these mice also displayed significantly decreased fed blood glucose levels, increased serum insulin levels and improved glucose tolerance, without changes in body weight, fasting glucose or insulin sensitivity (Fig. 2i–p). These findings from the β*Thada*-KO are completely consistent with the metabolic effects observed for the global *Thada* knockout and indicate that impacts from *Thada*-KO are mediated by β-cells.

### *Thada* knockout promotes pancreatic β-cell function in mice

**Fig. 3:**
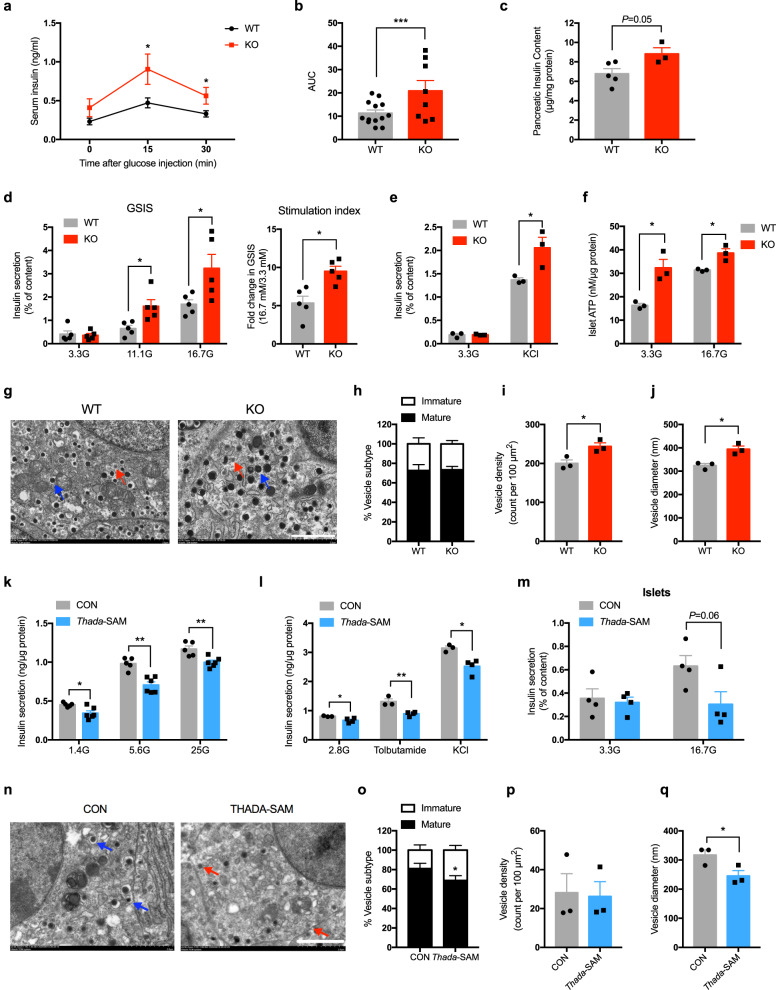
*Thada* knockout promotes pancreatic β-cell function in mice. **a** Serum insulin levels at 0, 15, and 30 min after intraperitoneal glucose injection in WT (*n* = 13) and *Thada*-KO mice (*n* = 8). **b** Area under the curve in (**a**). **c** Pancreatic insulin contents of WT (*n* = 5) and *Thada*-KO mice (*n* = 3). **d** Islets isolated from WT and *Thada*-KO mice were stimulated with 3.3, 11.1, or 16.7 mM glucose for 1 h and insulin secretion was assayed. Stimulation index was calculated as fold change of GSIS (*n* = 5). **e** Islets isolated from WT and *Thada*-KO mice were stimulated with or without 35 mM KCl at 3.3 mM glucose for 1 h and insulin secretion was assayed (*n* = 3). **f** ATP content of islets from WT and *Thada*-KO mice incubated at 3.3 or 16.7 mM glucose for 1 h (*n* = 3). **g** Transmission electron microscopy of pancreatic islets from WT and *Thada*-KO mice. Red and blue arrowheads point to vesicles containing immature and mature granules, respectively. Scale bar, 2 μm. **h**–**j** Analysis of ultrastructural β-cells from WT and *Thada*-KO mice (*n* = 3), including quantification of the percentage of mature and immature vesicles (**h**), vesicle density (**i**), and vesicle diameter (**j**). **k** MIN6 beta-cell lines were transduced with dCas9-SAM lentiviruses to activate *Thada* expression, then GSIS was assayed at 1.4 mM, 5.6 mM, and 25 mM glucose (*n* = 5 for control cells and *n* = 6 for *Thada*-SAM cells). **l** Control (CON, *n* = 3) and *Thada*-activated (*Thada*-SAM, *n* = 4) MIN6 beta-cell lines were stimulated at 2.8 mM glucose with or without 2.5 mM tolbutamide and 35 mM KCl for 1 h and insulin secretion was assayed. (**m**) Islets transduced with dCas9-SAM lentiviruses for 7 days were then stimulated with 3.3 and 16.7 mM glucose for 1 h and insulin secretion was assayed (*n* = 4). **n** Transmission electron microscopy of control and *Thada*-SAM MIN6 beta-cell lines. Red and blue arrowheads point to vesicles containing immature and mature granules, respectively. Scale bar, 2 μm. **o**–**q** Quantification of the percentage of mature and immature vesicles (**o**), vesicle density (**p**), and vesicle diameter (**q**) in control and *Thada*-SAM MIN6 beta-cell lines (*n* = 3). The animal experiments were performed on male mice at 8–10 weeks of age and female mice data were provided in Fig. S3. Data are presented as mean ± SEM. *P < 0.05, **P < 0.01, ***P < 0.001; significance is assessed by two-tailed unpaired Student’s *t* test. Source data are provided as a Source Data file.

We measured serum insulin levels after glucose challenge to examine if the metabolic effects in *Thada*-KO mice are related to altered insulin secretion. Indeed, *Thada* knockout led to dramatically increased serum insulin release after intraperitoneal glucose injection (Figs. 3a, b, S2p, S3a), along with increased pancreatic insulin content (Fig. 3c and S3b). To further assess the influence of THADA on β-cell function, primary islets isolated from WT and *Thada*-KO mice were treated ex vivo with different concentrations of glucose. Although basal insulin secretion at 3.3 mM glucose was comparable, the 11.1 and 16.7 mM glucose both stimulated significantly more insulin secretion from *Thada*-KO islets than WT islets, along with elevated stimulation index of *Thada*-KO islets (Fig. 3d).

We also found that KCl-stimulated insulin secretion was significantly increased in *Thada*-KO islets (Fig. 3e), suggesting that THADA modulates insulin release through a pathway downstream of glucose-induced inactivation of K_ATP_ channels. We then measured intracellular ATP content and detected significantly elevated ATP levels in *Thada*-KO islets (Fig. 3f). These results were substantiated in female *Thada*-KO mice (Fig. S3c–e). Further electron micrographs showed an unaltered proportion of mature insulin vesicles (Fig. 3g, h) but significantly increased overall number and mean size of the secretory vesicles in *Thada*-KO β-cells (Fig.3i, j), findings implicating THADA in the secretion of insulin vesicles in islet β-cells.

The CRISPR/catalytically-deficient Cas9 (dCas9)-synergistic activation mediator (SAM) system represents a powerful approach for selectively activating the transcription of endogenous genes^19^. We used this to activate *Thada* transcription in MIN6 cells, a commonly used mouse β-cell line, and detected stable up-regulation of *Thada* expression (~2.5 average fold-increase over control cells) (Fig. S3f, g). Note that this increase is quite close in magnitude to the observed THADA induction in T2DM patients and mice (Fig. 1). dCas9-SAM-mediated *Thada* activation led to significantly reduced insulin secretion elicited at various concentrations of glucose (Fig. 3k), and also decreased the insulin secretion stimulated by the depolarizing stimuli KCl and tolbutamide (Fig. 3l). Reduced glucose-stimulated insulin secretion (GSIS) was similarly observed in intact mouse islets in which *Thada* was activated with dCas9-SAM (Fig. 3m). Finally, we analyzed β-cell ultrastructure and found a significantly increased proportion of immature vesicles after *Thada* activation (Fig. 3n, o). Although the overall number of insulin vesicles remains unaffected (Fig. 3p), the mean size of insulin secretory vesicle was significantly declined after *Thada* activation (Fig. 3q). Consistently, both *Ins1* and *Ins2* transcriptions were down-regulated in *Thada*-activated cells (Fig. S3h). These findings collectively demonstrate that THADA impairs insulin production and secretion in β-cells.

### Increased β-cell mass owing to reduced apoptosis in *Thada*-knockout mice

**Fig. 4:**
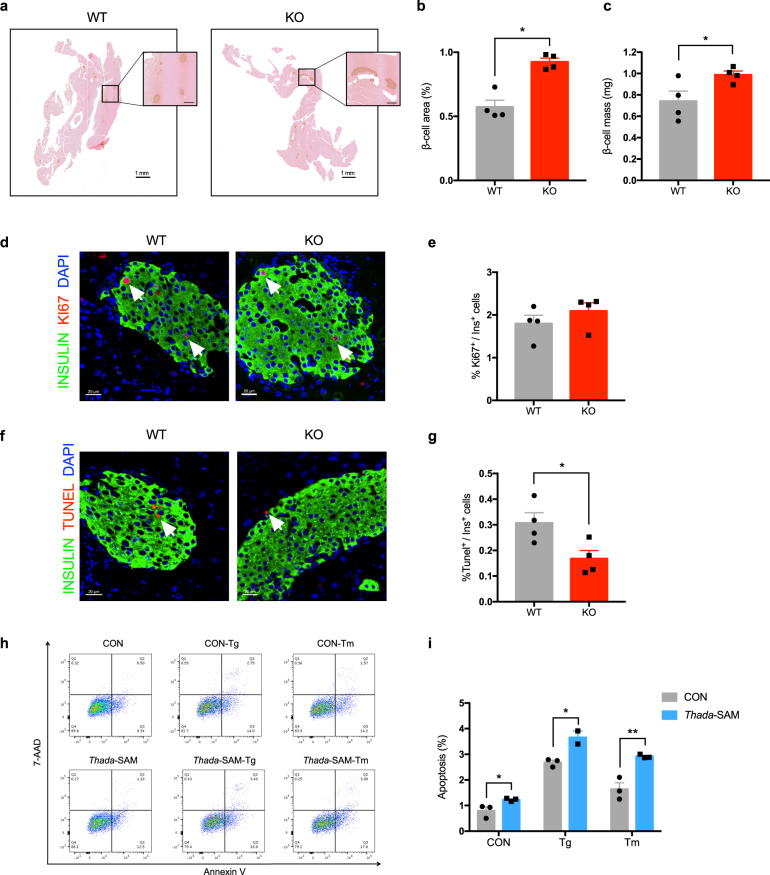
*Thada*-knockout mice have increased β-cell mass owing to reduced apoptosis. **a** Representative immunohistochemistry images of pancreatic sections from WT and *Thada*-KO mice stained for insulin (brown) and eosin (red). Scale bars, 1 mm for whole pancreas and 200 μm for magnified image. **b** Measurements of β-cell area/pancreatic area ratio in WT and *Thada*-KO mice (*n* = 4). **c** Measurements of β-cell mass in WT and *Thada*-KO mice (*n* = 4). **d** Representative immunofluorescence images of islets from WT and *Thada*-KO mice stained for insulin (green), Ki67 (red), and DAPI (blue). Scale bars, 20 μm. Arrowhead points to Ki67^+^Insulin^+^ cells. **e** The proliferation of β-cell was determined by quantification the percentage of Ki67^+^ in Insulin^+^ cells (*n* = 4 mice each group). 3428 ± 600 Insulin^+^ cells were quantified for WT mice and 3328 ± 533 Insulin^+^ cells were quantified for KO mice. **f** Representative immunofluorescence images of islets from WT and *Thada*-KO mice stained for insulin (green), Tunel (red), and DAPI (blue). Scale bars, 20 μm. Arrowhead points to Tunel^+^Insulin^+^ cells. **g** The apoptosis of β-cell was determined by quantification the percentage of Tunel^+^ in Insulin^+^ cells (*n* = 4 mice each group). 4204 ± 871 Insulin^+^ cells were quantified for WT mice and 4568 ± 591 Insulin^+^ cells were quantified for KO mice. **h** Flow cytometry analysis of control and *Thada*-SAM MIN6 beta-cell lines treated with or without 1 μM thapsigargin (Tg) or 1 μg/ml tunicamycin (Tm) for 24 h. Representative dot plots of cell apoptosis were shown after dual staining with Annexin V and 7-AAD. The gating strategy was provided in Fig. S4l. **i** The apoptosis of control and *Thada*-SAM MIN6 beta-cell lines were quantified by the percentage of Annexin V-positive cells (*n* = 4). The animal experiments were performed on male mice at 8-10 weeks of age and female mice data were provided in Figure S4. Data are presented as mean ± SEM. *P < 0.05, **P < 0.01; significance is assessed by two-tailed unpaired Student’s *t* test. Source data are provided as a Source Data file.

To test whether THADA also affects β-cell mass, we stained against insulin in pancreatic sections from WT and *Thada*-KO mice (Fig. 4a). Notably, both the ratio of β-cell area/pancreatic area and β-cell mass were significantly increased in *Thada*-KO mice (Fig. 4b, c), while overall islet architecture and the ratio of β-cells/α-cells were not perturbed (Fig. S4a, b). In addition, *Thada*-KO didn’t cause any significant difference in the percentage of α-cell area, α-cell mass, or serum glucagon levels (Fig. S4c–e). Ki67 and insulin dual-stainings of the pancreas showed no significant difference in β-cell proliferation (Fig. 4d, e), but we observed a striking reduction in the percentage of the apoptotic Tunel^+^Insulin^+^ cells in *Thada*-KO islets (Fig. 4f, g). Female *Thada*-KO mice showed the same tendency towards increased β-cell mass and less Tunel-positive β-cells as male mice, without change of β-cell proliferation (Fig. S4f–i).

To explore the role of THADA on β-cell survival, we performed CCK-8 assay and found dCas9-SAM-mediated *Thada* activation caused a significant reduction in β-cell viability (Fig. S4j). Although cell proliferation was not altered (Fig. S4k), flow cytometry showed significantly increased percentage of apoptotic β-cells after *Thada* activation (Fig. 4h, i). Further, given that the *Drosophila* THADA orthologue was reported to be an ER-resident protein^15^, it was interesting to note that this apoptosis induction was further potentiated upon exposure to thapsigargin (Tg) or tunicamycin (Tm), both were potent ER stress inducers (Figs. 4h, i, and S4l). Collectively, these results establish that beyond its impairment of insulin secretion, THADA can promote apoptosis and thereby induce loss of β-cell mass.

### THADA reduces ER calcium stores in β-cells

To investigate the molecular basis of the phenotypes caused by THADA, we profiled the transcriptome of *Thada*-activated MIN6 beta-cell line and detected the differentially expressed genes compared to control cells (Fig. S5a). Further Gene Set Enrichment Analysis (GSEA) revealed alteration of multiple biological processes related to β-cell function^20^ (Fig. S5b). Among these, the “positive regulation of release of sequestered calcium ion into cytosol”, “cytosolic calcium ion concentration involved in phospholipase C-activating G protein-coupled signaling pathway” and “phosphatidylinositol 3-kinase (PI3K) activity” were significantly enriched (Fig. S5c–e).

**Fig. 5:**
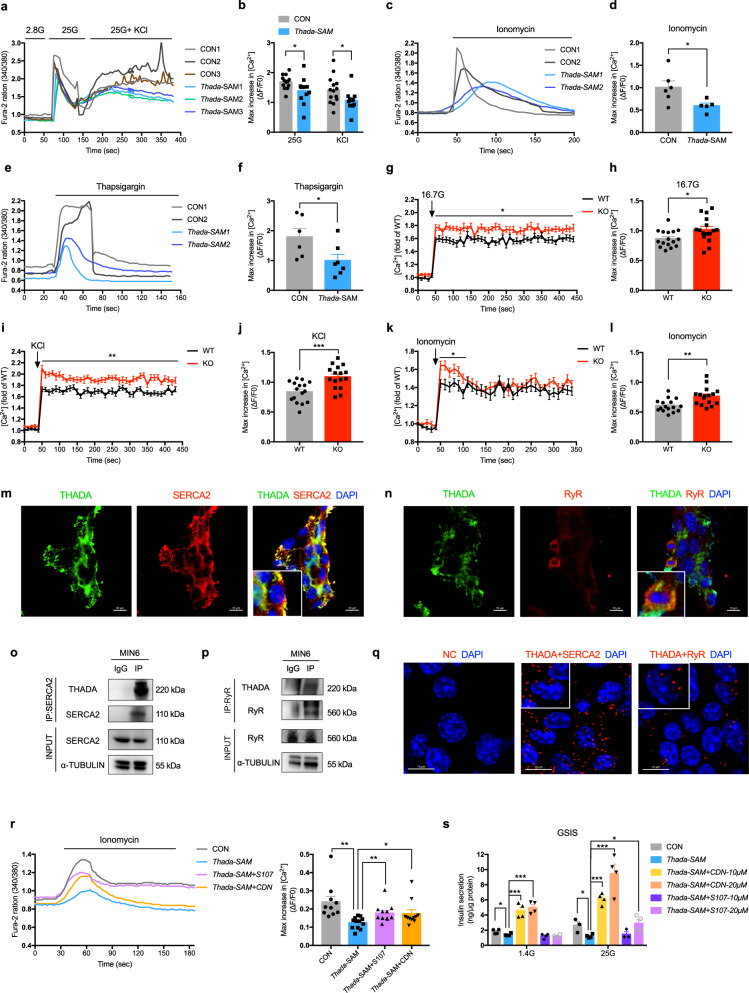
THADA reduces ER calcium stores and impairs insulin secretion through SERCA2 and RyR2 in β-cells. **a** Increases in [Ca^2+^]_i_ from control (CON) and *Thada*-activated (*Thada*-SAM) MIN6 beta-cell lines following stimulation with 2.8 mM glucose (2.8 G), 25 mM glucose (25 G) and 30 mM KCl. Representative results of three replicates from each group are provided. **b** Quantification of the maximum increases in [Ca^2+^]_i_ after 25 mM glucose (*n* = 13 for control and *n* = 11 for *Thada*-SAM) or 30 mM KCl stimulation. (*n* = 14 for control and *n* = 11 for *Thada*-SAM). **c**, **d** Quantification of the maximum increases in [Ca^2+^]_i_ from control (*n* = 6) and *Thada*-SAM β-cells (*n* = 5) after 10 μM ionomycin stimulation. Representative results of two replicates from each group are provided. **e**, **f** Quantification of the maximum increases in [Ca^2+^]_i_ from control (*n* = 6) and *Thada*-SAM β-cells (*n* = 7) after 2 μM thapsigargin stimulation. Representative results of two replicates from each group are provided. **g** [Ca^2+^]_i_ in dispersed islet cells from WT and *Thada*-KO mice at basal 2.8 mM glucose and after stimulation with 16.7 mM glucose (16.7 G) (*n* = 16 from six mice each group). **h** Quantification of the maximum increases in [Ca^2+^]_i_ after 16.7 mM glucose stimulation (*n* = 16 from six mice each group). **i**, **j** Quantification of [Ca^2+^]_i_ in dispersed islet cells from WT and *Thada*-KO mice after stimulation with 35 mM KCl at 2.8 mM glucose (*n* = 16 from six mice each group). **k**, **l** Quantification of [Ca^2+^]_i_ in dispersed islet cells from WT and *Thada*-KO mice after 10 μM ionomycin stimulation (*n* = 16 from six mice each group). **m** Immunofluorescence staining for THADA (green), SERCA2 (red), and DAPI (blue) in MIN6 beta-cell line. Scale bars, 10 μm. **n** Immunofluorescence staining for THADA (green), RyR (red), and DAPI (blue) in MIN6 beta-cell line. Scale bars, 10 μm. **o** MIN6 cells were immunoprecipitated with either a SERCA2 antibody or an IgG negative control, followed by western blot analysis using THADA and SERCA2 antibodies. **p** MIN6 cells were immunoprecipitated with either a RyR antibody or an IgG negative control, followed by western blot analysis using THADA and RyR antibodies. **q** Proximity ligation assay for THADA together with SERCA2 and RyR in MIN6 beta-cell line (NC for negative control). Scale bars, 10 μm. **r** Increases in [Ca^2+^]_i_ from control and *Thada*-activated β-cells pretreated with or without 20 μM S107 or 10 μM CDN1163 (CDN) after 10 μM ionomycin stimulation. Representative results from each group are presented (*n* = 10 for control, *n* = 12 for *Thada*-SAM, *n* = 11 for *Thada*-SAM with S107 and CDN1163). **s** Control and *Thada*-SAM β-cells were pretreated with or without CDN1163 and S107 at the indicated concentration for 24 h, then stimulated with 1.4 mM glucose (1.4 G) or 25 mM glucose for 1 h, and insulin secretion was assayed (*n* = 4, except for control cells at 25 G and *Thada*-SAM cells treated with 10 μM S107 at 25 G were *n* = 3). All western blots and immunostainings (m-q) show representative results from at least three independent experiments. Data are presented as mean ± SEM. *P < 0.05, **P < 0.01, ***P < 0.001; significance is assessed by two-tailed unpaired Student’s *t* test. Source data are provided as a Source Data file.

The calcium signaling is essential for β-cell function as elevated cytosolic Ca^2+^ directly triggers the exocytosis of insulin vesicles. We then performed calcium imaging experiments to assess whether THADA affects intracellular Ca^2+^ level ([Ca^2+^]_i_) in MIN6 β-cell line. No difference in the baseline [Ca^2+^]_i_ was observed (Fig. 5a and S5f), but upon stimulation with a 25 mM glucose load, *Thada*-activated cells exhibited a significantly blunted increase in [Ca^2+^]_i_ over control cells (Fig. 5a, b). Similarly, the [Ca^2+^]_i_ increase triggered by KCl was also significantly blunted in *Thada*-activated cells (Fig. 5a, b). Note that these results were consistent with the decreased glucose- and KCl-stimulated insulin secretion after *Thada* activation.

The ER is a major organelle for intracellular Ca^2+^ storage^21^. Our GSEA data implicating THADA in the release of sequestered calcium into cytosol (Fig. S5c) led us to evaluate β-cell ER Ca^2+^ levels. Ionomycin was used to deplete ER Ca^2+^ and thapsigargin was used to block Ca^2+^ re-uptake into the ER. Ionomycin induced a rapid and robust release of ER Ca^2+^ into cytosol in control cells (Fig. 5c). In contrast, *Thada*-activated cells displayed a much slower increase in [Ca^2+^]_i_ (Fig. 5c). Further, the maximal [Ca^2+^]_i_ increase was also reduced upon ionomycin stimulation (Fig. 5d). With thapsigargin, the increase in [Ca^2+^]_i_ was also significantly blunted in *Thada*-activated cells (Fig. 5e, f).

Next, we analyzed Ca^2+^ responses in primary islet cells. Consistent with increased GSIS, primary islet cells from *Thada*-KO mice exhibited significantly higher [Ca^2+^]_i_ in response to 16.7 mM glucose compared with cells from WT mice, without change in basal [Ca^2+^]_i_ (Fig. 5g, h). The increases in [Ca^2+^]_i_ triggered by KCl and ionomycin were also significantly enhanced in *Thada*-KO islet cells (Fig. 5i–l). As Inositol-1,4,5-trisphosphate (IP3)-dependent mobilization of ER Ca^2+^ is activated in response to glucose^22^, we examined IP3-induced Ca^2+^ release under high glucose condition and revealed significant increases in *Thada*-KO islet cells (Fig. S5g). Notably, the maximal increases in cytosolic Ca^2+^ after IP3 stimulation were comparable between WT and KO cells (Fig. S5h), suggesting THADA does not directly alter the activity of IP3 receptor (IP3R). We further used a CRISPR/Cas9 technique to generate *Thada*-KO MIN6 β-cell lines (Fig. S5i) and evaluated the ER Ca^2+^ levels of these cells. Compared to wild-type control cells, *Thada*-KO β-cells displayed significantly stronger increases in [Ca^2+^]_i_ upon both ionomycin and thapsigargin stimulations (Fig. S5j-m). These findings collectively support that THADA functions to reduce ER calcium stores in β-cells.

### THADA impairs insulin secretion through SERCA2 and RyR2

Ca^2+^ are sequestered within the ER through the activity of SERCA (Sarco/Endoplasmic Reticulum Ca^2+^-ATPases) and are released into cytosol following activation of IP3Rs and ryanodine receptors (RyRs)^23^, with RyR2 being the predominant form in β-cells (Fig. S6a). Guided by our results showing that THADA regulates ER Ca^2+^ stores, we hypothesized THADA’s effect may be mediated through these Ca^2+^ pump and/or channels residing in ER membrane. SERCA2 has been identified as the predominant isoform of ER Ca^2+^ pump in β-cells^24^. Immunofluorescence analyses of MIN6 β-cell lines revealed co-localization of THADA with SERCA2 and of THADA with RyR (Figs. 5m, n, S6b, c), while barely no co-localization signals of THADA with mitochondrial markers were detected (Fig. S6d, e). We also subjected MIN6 cell lysates to immunoprecipitaion (IP) with either an anti-SERCA2 antibody or an IgG negative control, and then probed the immunoprecipitated proteins with an anti-THADA antibody. Using this approach, we detected THADA protein as a ~220 kDa species among the immunoprecipitates (Fig. 5o). In similar experiments using an anti-RyR antibody, the immunoprecipitates also yielded a detectable THADA protein band (Fig. 5p). The physical interactions were further confirmed by proximity-ligation assays, which revealed that THADA exhibited proximity to SERCA2 and RyR in MIN6 beta-cell line (Fig. 5q). No altered phosphorylation of IP3R was observed after *Thada* activation, nor did any detectable binding of IP3R with THADA in MIN6 beta-cell line (Fig. S6f, g), indicating THADA has no direct impact on IP3R activity.

Previous work has shown that stress- or genetic mutation-induced dissociation of calstabin2 from RyR2, which stabilizes the closed state of the channel, results in ER Ca^2+^ depletion due to leaky RyR2 channel^25,26^. A small molecule drug, S107, could stabilize RyR2 and prevent ER Ca^2+^ leak^27^. Strikingly, treatment of *Thada*-activated β-cells with S107 restored the decreased ER Ca^2+^ level in these cells (Fig. 5r). The maximal [Ca^2+^]_i_ increase upon ionomycin stimulation in *Thada*-activated β-cells was also significantly increased by S107 (Fig. 5r). These results support that THADA’s interaction with RyR2 could cause the leakage of ER Ca^2+^. We then tested a known allosteric activator of the Ca^2+^ pump SERCA2—CDN1163^28^. Our finding that CDN1163 treatment significantly increased ER Ca^2+^ stores in *Thada*-activated β-cells (Fig. 5r) suggest that THADA inhibits Ca^2+^ re-uptake through SERCA2.

We then examined whether restoring the ER Ca^2+^ levels through SERCA2 and RyR2 could rescue THADA-induced β-cell functional deficit. Activation of SERCA2 by CDN1163 treatment dose-dependently enhanced GSIS in *Thada*-activated β-cells (Fig. 5s). Treatment with the RyR2 stabilizer S107 also restored THADA-decreased GSIS (Fig. 5s). Upon depolarizing KCl stimulation, S107 treatment also rescued THADA-decreased insulin secretion to control levels (Fig. S6h). Taken together, these results suggest that THADA reduces ER Ca^2+^ stores in β-cells by interacting with SERCA2 and RyR2, thus leading to impaired insulin secretion.

### THADA aggravates ER stress-induced apoptosis by activating DR5/FADD/caspase-8 in β-cells

**Fig. 6:**
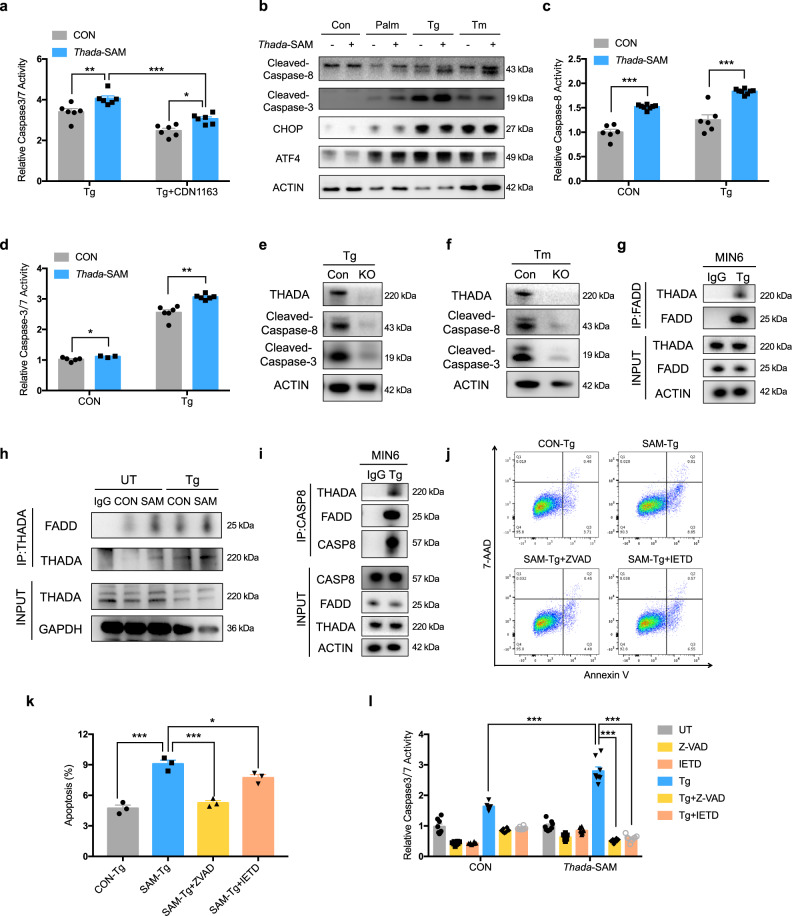
THADA aggravates ER stress-induced apoptosis by activating DR5/FADD/caspase-8 in β-cells. **a** The caspase-3/7 activities of control and *Thada*-SAM β-cells were assayed after treatment with 1 μM thapsigargin (Tg) in the presence or absence of 10 μM CDN1163 for 24 h (*n* = 6). **b** Control and *Thada*-SAM β-cells were treated with or without 0.5 mM palmitate (Palm), 1 μM thapsigargin or 1 μg/ml tunicamycin (Tm) for 24 h, then cleaved caspase-8, cleaved caspase-3, CHOP and ATF4 protein levels were determined. **c** The caspase-8 activities of control (*n* = 6) and *Thada*-SAM (*n* = 8) β-cells were assayed after treatment with or without 1 μM Tg for 24 h. **d** The caspase-3/7 activities of control and *Thada*-SAM β-cells were assayed after treatment with vehicle (*n* = 5 for control and *n* = 3 for *Thada*-SAM) or 1 μM Tg (*n* = 6) for 24 h. **e**, **f** Control and *Thada*-KO MIN6 beta-cell lines were treated with 1 μM Tg (**e**) or 1 μg/ml Tm (**f**) for 24 h, then cleaved caspase-8 and cleaved caspase-3 protein levels were determined. **g** MIN6 beta-cell lines were treated with 1 μM Tg for 24 h, then immunoprecipitated with either an IgG control or a FADD antibody, followed by western blot analysis with a THADA antibody. **h** Control and *Thada*-SAM cells were untreated (UT) or treated with 1 μM Tg for 24 h, then immunoprecipitated with either an IgG control or a THADA antibody, followed by western blot analysis with a FADD antibody. (**i**) MIN6 beta-cell lines were treated with 1 μM Tg for 24 h, then immunoprecipitated with either an IgG control or a caspase-8 antibody, followed by western blot analysis with THADA and FADD antibodies. **j** Flow cytometry analysis of control and *Thada*-SAM β-cells treated with 1 μM Tg in the presence or absence of 20 μM Z-VAD or 20 μM Z-IETD for 24 h. Representative dot plots of cell apoptosis were shown after dual staining with Annexin V and 7-AAD. **k** The apoptosis of control and *Thada*-SAM β-cells were quantified by the percentage of Annexin V-positive cells (*n* = 3). **l** The caspase-3/7 activities of control and *Thada*-SAM β-cells were assayed after treatment with or without 1 μM Tg in the presence or absence of 20 μM Z-VAD or 20 μM Z-IETD for 24 h (*n* = 8 biologically independent samples). All western blots show representative results from at least three independent experiments. Data are presented as mean ± SEM. *P < 0.05, **P < 0.01, ***P < 0.001; significance is assessed by two-way ANOVA followed by Tukey’s multiple comparison test (**a**, **l**), two-tailed unpaired Student’s *t* test (**c**, **d**), or one-way ANOVA followed by Dunnett’s multiple test (**k**). Source data are provided as a Source Data file.

As increased β-cell apoptosis was observed after *Thada* activation, we further explored the underlying mechanisms. ER Ca^2+^ depletion can result in ER stress and β-cell apoptosis^29^, so we initially checked whether restoring the ER Ca^2+^ levels may rescue THADA-induced apoptosis. *Thada* activation significantly potentiated Tg-increased caspase-3/7 activity; however, increasing the ER Ca^2+^ store based on CDN1163 treatment only partially suppressed caspase-3/7 activation (Fig. 6a), suggesting an alternative mechanism for THADA-induced apoptotic phenotype apart from the ER Ca^2+^ signaling. Persistent ER stress and the ensuing unfolded protein response (UPR) trigger apoptosis through the PERK-ATF4-CHOP axis^30^. Immunoblotting of ATF4 and CHOP levels in response to nutritional and ER stress-inducing agents showed that 24 h treatment of β-cells with palmitate, thapsigargin and tunicamycin induced robust increases in ATF4 and CHOP expression, indicative of activated UPR (Fig. 6b). However, we detected no differences in the levels of these two proteins between control and *Thada*-activated cells (Fig. 6b and S7a).

Persistent ER stress activates death receptor 5 (DR5) and then triggers apoptosis via caspase-8, the initiator caspase downstream of the death receptor pathway^31^. Interestingly, we detected significantly increased cleavage of caspase-8 and caspase-3 after *Thada* activation in response to various ER stress inducers (Fig. 6b and S7a). Additionally, *Thada* activation significantly increased both caspase-8 and caspase-3/7 activities, which were further enhanced upon Tg-induced ER stress (Fig. 6c, d). In contrast, ablation of *Thada* significantly suppressed Tg-induced apoptosis (Fig. S7b, c). THADA depletion also strongly inhibited the activities of both caspase-8 and caspase-3 under Tg- and Tm-induced ER stress conditions (Fig. 6e, f), suggesting that THADA aggravates caspase-8-initiated apoptosis.

As caspase-8 activation is controlled by DR5 under persistent ER stress^31^, we then tested whether THADA may promote caspase-8 activity via DR5. The DR5 protein was present in an anti-THADA immunoprecipitate upon Tg-induced ER stress in HeLa cells (Fig. S7d); conversely, THADA was present in an anti-DR5 immunoprecipitate from similarly stressed cells (Fig. S7e). DR5 activates caspase-8 via the “adaptor” protein Fas-associated death domain (FADD)^32^. Co-IP showed an interaction of THADA with FADD under persistent ER stress (Fig. S7f, g) and immunofluorescence showed co-localization of THADA with DR5 and with FADD (Fig. S7h).

We also tested the THADA-FADD interaction in MIN6 beta-cell line (Fig. 6g) and found that under ER stress conditions the THADA-FADD interaction was stronger in *Thada*-activated β-cells than in control cells (Fig. 6g, h). Moreover, co-IP of β-cells under Tg-induced ER stress revealed interaction between THADA and caspase-8 apoptotic complexes (Fig. 6i). To confirm a contribution from capase-8 in THADA-induced apoptosis, we treated *Thada*-activated β-cells with the pan-caspase inhibitor Z-VAD or the caspase-8 inhibitor Z-IETD. Both inhibitors significantly blocked THADA-aggravated apoptosis upon Tg-induced ER stress (Fig. 6j, k) and led to significantly decreased caspase-3/7 activities (Fig. 6l). Taken together, these results demonstrate that THADA aggravates ER stress-induced apoptosis by interacting and activating the pro-apoptotic complex of DR5/FADD/caspase-8 in β-cells.

We next explored whether *Thada* expression was controlled by ER stress. We detected up-regulated *Thada* transcription induced by diverse ER stressors in mouse islets (Fig. S7i). The protein level was also increased in MIN6 beta-cell line under these conditions (Fig. S7j). Persistent ER stress activates UPR, being evident by time-dependently increased *Atf4* and *Chop* transcriptions (Fig. S7k). In parallel, THADA expression was also activated by prolonged ER stressor treatment (Fig. S7k, l). Moreover, small interfering RNA (siRNA) knockdown of *Atf4* and *Chop* significantly blocked Tg-induced *Thada* transcription (Fig. S7m). These findings support that persistent ER stress activates THADA expression via the UPR mediator ATF4 and CHOP.

### *Thada* knockout protects mice from HFHS diet-induced glucose intolerance by improving β-cell function and suppressing β-cell apoptosis

**Fig. 7:**
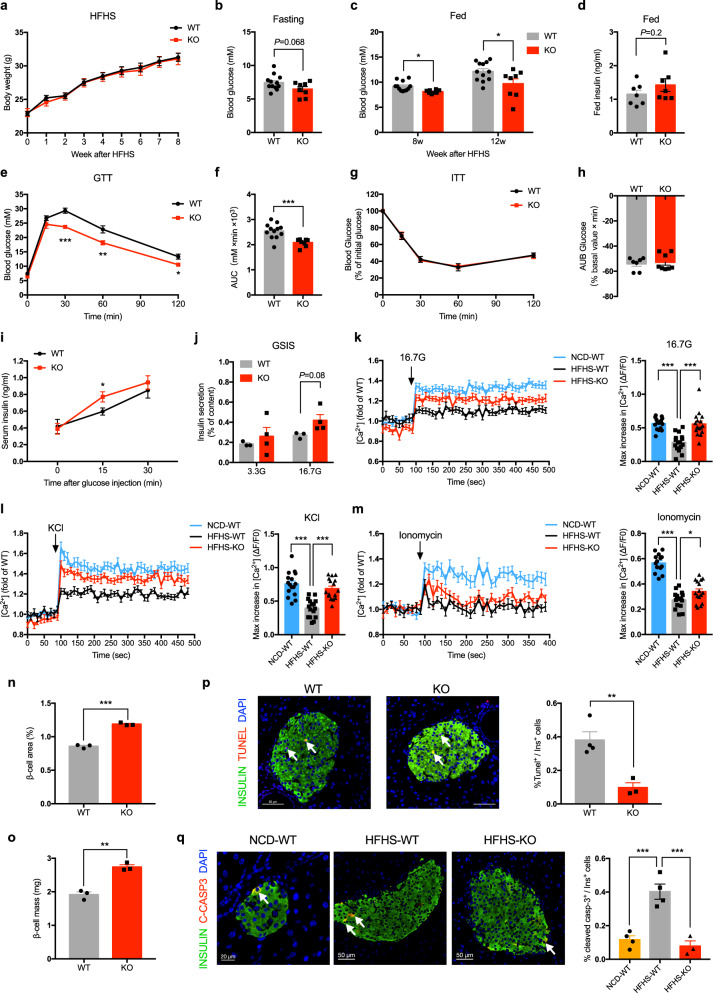
*Thada* knockout protects mice from HFHS diet-induced glucose intolerance by improving β-cell function and suppressing β-cell apoptosis. **a** Body weights of WT (*n* = 12) and *Thada*-KO mice (*n* = 8) after HFHS diet for the indicated weeks. **b** Blood glucose levels of HFHS-fed WT (*n* = 12) and *Thada*-KO mice that were fasted overnight (*n* = 8). **c** Fed blood glucose levels of WT (*n* = 12) and *Thada*-KO mice (*n* = 8) after HFHS diet for 8 and 12 weeks. **d** Fed serum insulin levels of HFHS-fed WT and *Thada*-KO mice (*n* = 7). **e**, **f** IPGTT and area under the curve of HFHS-fed WT (*n* = 12) and *Thada*-KO mice (*n* = 8). **g**, **h** ITT and area under baseline of HFHS-fed WT (*n* = 7) and *Thada*-KO mice (*n* = 8). **i** Serum insulin levels at 0, 15, and 30 min after intraperitoneal glucose injection in HFHS-fed WT and *Thada*-KO mice (*n* = 8 for 0 min, *n* = 12 for 15 and 30 min). **j** Islets isolated from HFHS-fed WT (*n* = 3) and *Thada*-KO mice (*n* = 4) were stimulated with 3.3 and 16.7 mM glucose for 1 h, and insulin secretion was assayed. **k** Quantification of [Ca^2+^]_i_ in primary islet cells from NCD-fed WT, HFHS-fed WT and HFHS-fed *Thada*-KO mice at basal 2.8 mM glucose and after stimulation with 16.7 mM glucose (*n* = 16 from five mice each group). **l** Quantification of [Ca^2+^]_i_ in primary islet cells from NCD-fed WT, HFHS-fed WT and HFHS-fed *Thada*-KO mice at basal 2.8 mM glucose and after stimulation with 35 mM KCl (*n* = 16 from five mice each group). **m** Quantification of [Ca^2+^]_i_ in primary islet cells from NCD-fed WT, HFHS-fed WT and HFHS-fed *Thada*-KO at basal 5.6 mM glucose and after 10 μM ionomycin stimulation (*n* = 14 for NCD-WT, *n* = 15 for HFHS-WT, *n* = 16 for HFHS-KO from five mice each group). **n** Measurements of β-cell area/pancreatic area ratio in HFHS-fed WT and *Thada*-KO mice (*n* = 3). **o** Measurements of β-cell mass in HFHS-fed WT and *Thada*-KO mice (*n* = 3). **p** Representative images of islets from HFHS-fed WT and *Thada*-KO mice stained for insulin (green), Tunel (red), and DAPI (blue). Scale bars, 50 μm. Arrowhead points to Tunel^+^Insulin^+^ cells. The percentage of Tunel^+^ in Insulin^+^ cells was quantified (*n* = 4 mice). At least 3000 Insulin^+^ cells in each mouse were counted for quantification. **q** Representative images of islets from NCD-fed WT, HFHS-fed WT, and HFHS-fed *Thada*-KO mice stained for insulin (green), cleaved caspase-3 (red), and DAPI (blue). Scale bar was 20 or 50 μm as indicated. Arrowhead points to cleaved caspase-3^+^Insulin^+^ cells. The percentage of cleaved caspase-3^+^ in Insulin^+^ cells was quantified (*n* = 4 mice for NCD-WT and HFHS-WT, *n* = 3 mice for HFHS-KO). At least 2500 Insulin^+^ cells in each mouse were counted for quantification. The experiments were performed on male mice after HFHS diet for 12 weeks unless otherwise indicated and female mice data were provided in Fig. S8. Data are presented as mean ± SEM. *P < 0.05, **P < 0.01, ***P < 0.001; significance is assessed by two-tailed unpaired Student’s *t* test (a–**j**, **n**–**p**) or one-way ANOVA followed by Dunnett’s multiple comparison test (**k**, **l**, **m**, **q**). Source data are provided as a Source Data file.

Chronic nutrient overload is a main driver for T2DM. Recalling our finding that loss of THADA led to improved glucose homeostasis in mice (Fig. 2), we hypothesized that THADA deficiency might protect mice from diet-induced hyperglycemia. Pursuing this, we fed WT and *Thada*-KO mice a high-fat high-sucrose (HFHS) diet for 12 weeks. There were no differences in the body weights or fasting blood glucose levels of the two genotypes (Fig. 7a, b). The HFHS diet-induced elevated fed blood glucose levels in WT mice, reaching an average of 12.2 mM after feeding for 12 weeks (Fig. 7c). In contrast, *Thada*-KO mice exhibited significantly lower fed blood glucose than their WT littermates (Fig. 7c). Though fed insulin only showed a trend towards increase (Fig. 7d), *Thada*-KO mice displayed significantly improved glucose tolerance compared to their WT littermates after HFHS feeding for 12 weeks (Fig. 7e, f), without change in insulin sensitivity (Fig. 7g, h). *Thada*-KO also substantially protected female mice from HFHS diet-induced hyperglycemia and glucose intolerance (Fig. S8a–h).

Further experiments showed that HFHS-fed *Thada*-KO mice had significantly higher serum insulin releases post glucose challenge compared to HFHS-fed WT mice (Fig. 7i and S8i), helping to explain the improved glucose tolerance observed in these mice. As a direct measure of β-cell function, GSIS was assessed with isolated islets: under high glucose stimulation at 16.7 mM, islets from HFHS-fed *Thada*-KO mice secreted more insulin than islets from WT mice (Fig. 7j). In line with this, assessment of Ca^2+^ responses revealed that islet cells from HFHS-fed *Thada*-KO mice exhibited significantly higher [Ca^2+^]_i_ in response to 16.7 mM glucose and 35 mM KCl compared with cells from WT mice (Fig. 7k, l). The maximal [Ca^2+^]_i_ increase was also significantly enhanced upon ionomycin stimulation in *Thada*-KO islet cells (Fig. 7m). These data collectively support that ablation of *Thada* improved β-cell function by preserving ER Ca^2+^ stores under HFHS diet.

*Thada*-KO mice displayed significant increases in the ratio of β-cell area/pancreatic area (Fig. 7n), and their β-cell mass was significantly increased (Fig. 7o). We also detected markedly reduced β-cell apoptosis in these *Thada*-KO mice (Fig. 7p). Further, HFHS diet-induced β-cell caspase-3 activation was blocked by *Thada* KO (Fig. 7q), along with reduced cleaved caspase-8-positive β-cells and caspase-8 activity in *Thada*-KO islets (Fig. S8j–l), indicating *Thada* depletion suppressed HFHS diet-induced β-cell apoptosis via caspase-8.

To test the direct therapeutic effect of THADA on rescuing β-cells, we isolated islets from long-term HFHS diet-fed mice and infected them with lentivirus encoding Cas9-control or Cas9-sgRNA targeting *Thada*. We found that ablation of *Thada* significantly restored the impaired insulin secretion in islets from HFHS-fed mice (Fig. S8m). Whereas islets from HFHS-fed mice showed increased caspase-8 and caspase-3 cleavage, this was dramatically reduced by *Thada* knockout (Fig. S8n). Thus, THADA deficiency confers therapeutic benefits by improving β-cell function and suppressing β-cell apoptosis.

### *Thada* knockout protects mice from HFD/STZ-induced hyperglycemia and β-cell loss

We induced high-fat diet (HFD)/STZ T2DM mouse model^33^ to further investigate the impacts of *Thada* knockout on glycemic control. *Thada*-KO and WT littermate control mice were fed a HFD and then injected with a single dose of STZ (Fig. S9a). No difference in body weight was observed between model animals of these two genotypes (Fig. S9b). By day 14 after the STZ injection, WT mice had developed severe hyperglycemia, whereas *Thada*-KO mice displayed lower 6-h fasting blood glucose levels (Fig. S9c). *Thada*-KO mice also showed significantly decreased fed blood glucose levels compared with WT mice (Fig. S9d), and their serum insulin levels were elevated (Fig. S9e). As expected, *Thada* knockout significantly improved the glucose tolerance in mice (Fig. S9f, g). Female HFD/STZ *Thada*-KO mice exhibited similar phenotypes as male mice, showing markedly decreased hyperglycemia and improved glucose tolerance compared with WT littermates (Fig. S9h–l).

We also examined whether *Thada* knockout protects against β-cell loss after HFD/STZ induction. Indeed, the HFD/STZ *Thada*-KO mice displayed a 1.46-fold increase in the ratio of β-cell area/pancreatic area over the WT mice (Fig. S9m). The β-cell mass of *Thada*-KO mice was also 1.6-fold higher than the WT mice (Fig. S9n). Tunel staining to assess apoptosis revealed a 35% reduction in Tunel-positive β-cells in *Thada*-KO mice compared with WT littermates (Fig. S9o). We also detected an obvious reduction in cleaved caspase-8-positive β-cells in *Thada*-KO mice (Fig. S9p). Collectively, these results provide additional support for the idea that suppressing β-cell apoptosis and preserving β-cell mass by disrupting THADA function can afford significant improvements in glycemic control and protect against development of T2DM.

### A high-content screen identifies alnustone that can reverse THADA-induced β-cell dysfunction and ameliorate hyperglycemia in obese mice

**Fig. 8:**
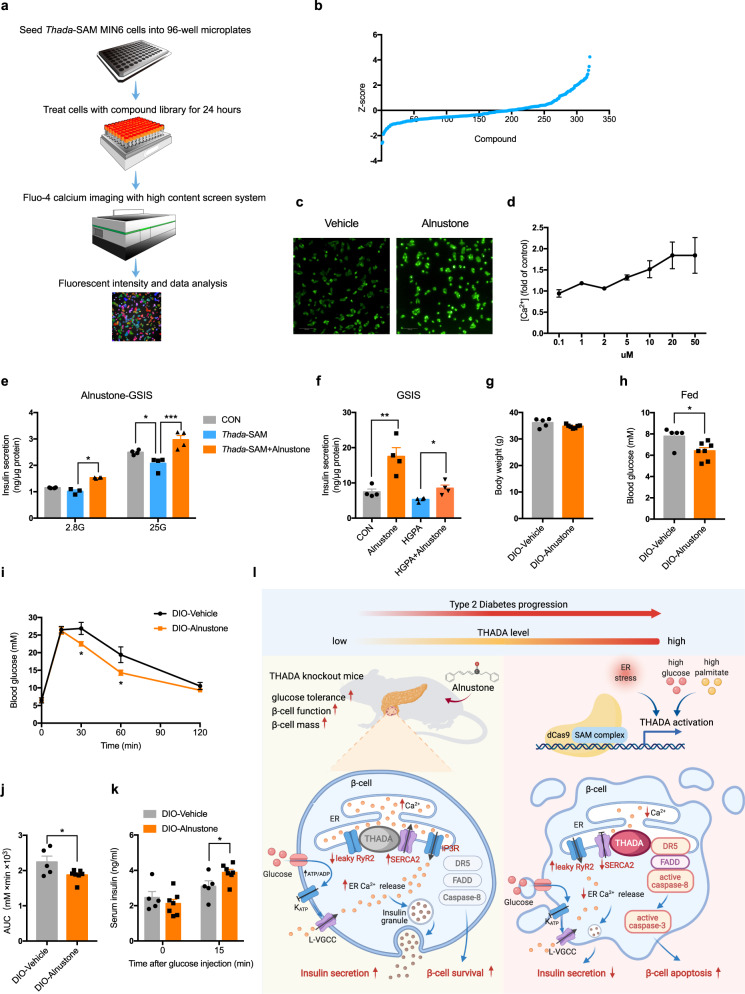
A high-content screen identifies alnustone that can reverse THADA-induced β-cell dysfunction and ameliorate hyperglycemia in obese mice. **a** A high-content screening workflow to identify compounds that can reverse THADA-reduced [Ca^2+^]_i_. **b** Primary screen data. Glucose-stimulated [Ca^2+^]_I_ was normalized to *Z* score. **c** Representative images of glucose-stimulated fluo-4 Ca^2+^ fluorescence after treatment with vehicle or alnustone for 24 h. Scale bar, 100 μm. **d** Dose curve of alnustone on the glucose-stimulated [Ca^2+^]_i_ (*n* = 3 biologically independent experiments). **e** GSIS of *Thada*-SAM β-cells treated with or without 10 μM alnustone for 24 h (*n* = 4 biologically independent experiments). **f** MIN6 beta-cell lines were treated with or without 33 mM glucose and 0.5 mM palmitate (HGPA) in the presence or absence of 10 μM alnustone for 24 h. Then GSIS was assayed (*n* = 4 biologically independent experiments). **g**–**k** DIO male mice were injected with vehicle or 10 mg/kg alnustone for 5–7 days, then body weights (**g**), fed blood glucose levels (**h**), IPGTT and the related AUC (**i**, **j**) as well as serum insulin levels at 0 and 15 min after intraperitoneal glucose injection (**k**) were measured (*n* = 5 for vehicle and *n* = 7 for alnustone). **l** Model depicting the role of THADA in the pathogenesis of T2DM: THADA expression is induced by glucolipotoxicity in β-cells during T2DM progression, which leads to ER Ca^2+^ depletion due to inhibited SERCA2 activity and leaky RyR2 Ca^2+^ channel, and activation of the DR5/FADD/caspase-8 pro-apoptotic complex, consequently resulting in impaired insulin secretion and aggravated ER stress-induced β-cell apoptosis. Genetic ablation or inhibition of THADA by alnustone improves glucose tolerance in mice by promoting β-cell function and survival. Image was created with BioRender.com. K_ATP_: ATP-sensitive K^+^ channel; L-VGCC: L-type voltage-gated Ca^2+^ channel; ER: endoplasmic reticulum; SAM: synergistic activation mediator. Data are presented as mean ± SEM. *P < 0.05, **P < 0.01, ***P < 0.001; significance is assessed by two-way ANOVA followed by Tukey’s multiple comparison test (**e**) or two-tailed unpaired Student’s *t* test (**f**–**k**). Source data are provided as a Source Data file.

To identify potential therapeutic drug candidates for T2DM, we developed a high-content screening assay targeting THADA’s function in reducing [Ca^2+^]_i_. *Thada*-activated β-cells were treated with a compound library containing FDA-approved drugs as well as traditional Chinese medicine monomers for 24 h. After loading with fluo-4 Ca^2+^ indicator, these cells were further stimulated with 25 mM glucose and imaged for fluorescence. Then glucose-stimulated [Ca^2+^]_i_ was quantified (Fig. 8a). From 320 compounds, six compounds that increased the *Z* score to >2.5 were defined as primary hits (Fig. 8b), which were further validated by dose-response and effects on insulin secretion. After confirmation, two compounds, proanthocyanidin and alnustone, were identified to have the most striking effects (Fig. S10a).

Proanthocyanidins are widely present in dietary fruits and foods, known for their strong antioxidant properties and beneficial effects on pancreatic β-cell function^34^. We found that this compound increased glucose-stimulated [Ca^2+^]_i_ in a dose-dependent manner in *Thada*-activated cells (Fig. S10b, c). Importantly, it significantly reversed THADA- as well as high glucose and palmitate-impaired insulin secretion (Fig. S10d, e), confirming the potency of this screening strategy to identify potential therapeutic candidates by targeting THADA.

Alnustone is a natural compound found within extracts of *Alpinia katsumadai* Hayata. Although it was reported to exhibit anti-inflammatory activity^35^, we are unaware of any reports that alnustone modulates insulin secretion or glucose homeostasis. Here we revealed that alnustone could dose-dependently increase glucose-stimulated [Ca^2+^]_i_ in *Thada*-activated cells (Fig. 8c, d). It significantly potentiated GSIS and completely reversed THADA- as well as high glucose and palmitate-impaired insulin secretion in β-cells (Fig. 8e, f). Alnustone treatment also suppressed Tg-induced caspase-8 and caspase-3/7 activations (Fig. S10f, g). Moreover, systemic alnustone administration to HFD-induced obese (DIO) mice significantly decreased fed blood glucose and improved glucose tolerance, without adverse effect on body weight (Fig. 8g–j). This was concomitant with significantly increased serum insulin release after glucose challenge (Fig. 8k), without change in insulin sensitivity (Fig. S10, i). Alnustone also exhibited effective anti-hyperglycemic effect in DIO female mice (Fig. S10j–m). Together, these results support that alnustone is a promising lead compound in the pursuit of a pharmacological approach for the treatment of T2DM.

## Discussion

In the present study, we proposed a working model to delineate how THADA modulates β-cell function and mass to participate in the pathogenesis of T2DM (Fig. 8m). Chronically elevated glucolipotoxicity drives gradual increases in islet THADA expression. In prediabetic phase, THADA activation decreases ER Ca^2+^ stores through SERCA2 and RyR2, thereby impairing the insulin secretory function of β-cells. Uncontrolled hyperglycemia can then exacerbate β-cell ER stress, under which condition THADA aggravates ER stress-induced apoptosis through the pro-apoptotic complex of DR5/FADD/caspase-8. This culminates in the loss of β-cell mass and causes severe hyperglycemia. Interfering in this THADA-mediated β-cell failure process could protect against the development of T2DM.

A surprising finding from our study was that ablation of THADA is beneficial for whole-body glycemic control, which resonates with the previously reported observation that a T2DM-associated *THADA* variant is correlated with random blood glucose levels^36^. Of note, *THADA* risk variants for PCOS—a female reproductive disorder that is accompanied by insulin resistance in 44%-70% of cases^37^—have also been linked to fasting blood glucose and metabolic syndrome^38,39^. Given that women with PCOS have a substantially increased incidence of T2DM^40^, our findings suggest that aberrant THADA function may underlie the shared pathophysiology of glucose metabolic disturbance for T2DM and PCOS.

Our results also support that the beneficial effect of THADA deficiency on glucose homeostasis results from an enhancement of β-cell function and preservation of β-cell mass. Thomsen et al. reported that siRNA-mediated silencing of *THADA* in a human β-cell line resulted in elevated insulin secretion, both under basal glucose and in response to IBMX and tolbutamide^41^. Those in vitro results are in agreement with our in vivo finding that THADA deficiency improves β-cell function. Islet has been proposed to be the primary tissue that contributes to T2DM-associated genetic signal of *THADA*, with fewer contributions from liver, adipose and muscle^42^. Supporting this, beta-cell-specific *Thada*-KO mice phenocopies the global knockout mice (although Cre-containing mice would be better controls in the conditional knockout study). In addition, we did not observe altered body weights or insulin sensitivity in global *Thada*-KO mice. The discrepancy between our results and the obese phenotype of *THADA*-KO *Drosophila*^15^ could be due to more complex and orchestrated metabolic regulation in mammals. Therefore the mild thermogenic phenotype caused by *Thada* deletion might be compensated in mice. Overall, our work strongly supports THADA as a crucial determinant for pancreatic β-cells to control systemic glucose homeostasis.

The ER serves as the largest intracellular Ca^2+^ pool, which is tightly controlled by the Ca^2+^ pump SERCA2 as well as by Ca^2+^ channels including IP3R and RyRs^43^. Our data reveals that THADA interacting with SERCA2 and RyR2 prevents ER Ca^2+^ re-uptake and causes leaky RyR2 channel, whereas THADA deficiency increases ER Ca^2+^ levels. Following glucose metabolism in β-cell, the ATP-sensitive K_ATP_ channels are closed and L-type voltage-gated Ca^2+^ channels in the plasma membrane are opened. The ensuing extracellular Ca^2+^ influx into cytosol activates phospholipase C and then generates IP3, which further induces Ca^2+^ release from the ER through activating IP3R^22,44^. Cytosolic Ca^2+^ also stimulates RyRs and leads to “Ca^2+^-induced Ca^2+^ release”^45^. We found *Thada*-KO β-cells exhibited significantly elevated IP3-induced Ca^2+^ releases, supporting for the increased ER Ca^2+^ stores. This ultimate rise in cytosolic Ca^2+^ drives more insulin release in *Thada*-KO β-cells. To be noted, GWAS has revealed an association between *RYR2* and T2DM^46^. Reduced islet SERCA2 expression was also detected in several T2DM models^24^. *Ryr2* mutation mice displayed decreased insulin release and impaired glucose homeostasis due to the leakage of Ca^2+^ from ER^27^ (despite the low abundance of RyRs in beta-cells). Similarly, mice with global *Serca2* heterozygosity exhibit hyperglycemia in response to HFD owing to impaired insulin biosynthesis and secretion, but have normal adiposity and insulin sensitivity^47^. The β-cell functional deficits in these mice with depleted ER Ca^2+^ stores are consistent with the effects of *Thada* activation. As insulin gene transcription was inhibited by *Thada* activation, the impaired insulin biogenesis could account for the aberrant density and size of insulin vesicles. Downstream signals in insulin and calcium pathways may also contribute to the alteration of insulin vesicles. Apart from these, it’s also possible that THADA could interact with and impact other molecules that are involved in insulin vesicle formation or the exocytosis machinery, although the underlying molecular mechanisms will need to be further investigated in the future.

In addition to storing Ca^2+^, ER is a main site for the quality control of secretory proteins. Dysregulation of this quality control processes triggers ER stress and activates UPR. In the face of manageable ER stress, adaptive UPR increases the functional capacity of ER to make sure that secretory client proteins, such as proinsulin, become properly folded. However, if irremediable ER stress persists, the “double-edged” UPR ultimately commits cells to apoptosis^48^, leading to β-cell loss in T2DM. Intriguingly, we found THADA expression was induced in β-cells when ER stress persists, indicating it is involved in the pro-apoptotic pathway instead of the compensatory UPR. UPR signals converge on DR5 and induce the formation of caspase-8-activating complex (comprising caspase-8, DR5 and the caspase-8 adaptor FADD) to drive ER stress-induced apoptosis^31,49^. Misfolding of insulin has also been shown to induce cell apoptosis in a DR5-dependent manner^50^. We revealed that THADA-induced apoptosis was aggravated through DR5/FADD/caspase-8 signaling upon exposure of β-cells to chronic ER stress. Our data further this concept by experimentally confirming that THADA aggravates caspase-8-initiated apoptosis in response to ER stress by interacting with the caspase-8-activating complex. Thus, our results indicate that THADA regulates the life-vs-death decisions of β-cells upon chronic ER stress.

Patients with T2DM are characterized by elevated glucose and free fatty acid levels in circulation. This glucolipotoxic condition is known to contribute to impaired β-cell function and ultimately drive β-cell failure^51^. Interestingly, blood-based analyses of T2DM patients revealed elevated *THADA* transcription^52^, consistent with our observation that THADA was up-regulated in islets of T2DM patients as well as *db/db* mice and HFHS-fed mice (when they had developed overt diabetes). By exploiting a high-content screen targeting THADA’s function in reducing [Ca^2+^]_i_, we further identified a natural compound, alnustone, capable of improving β-cell function and alleviating hyperglycemia in DIO mice. Verification of this anti-diabetic effect of alnustone paves way for developing an effective strategy targeting THADA for T2DM therapies.

In summary, the present study shed light onto the pathophysiological role of THADA in T2DM by controlling both β-cell function and apoptosis. Our results provide insights and scientific basis to support that inhibiting THADA expression and/or activity could be a promising intervention strategy for the prevention and treatment of T2DM.

## Methods

### Generation of global and β-cell-specific *Thada* knockout mice

Global *Thada*-knockout mice were generated using a CRISPR/Cas9-mediated genome editing system from Cyagen Biosciences (Guangzhou, China). Briefly, *Cas9* mRNA and dual sgRNAs targeting exon 8 to exon 11 of mouse *Thada* gene were generated by in vitro transcription, and then microinjected into the fertilized eggs from C57BL/6 mice. The founder mice were confirmed by genotyping and DNA sequencing analysis, with positive founders bred to the next generation. The PCR primers for *Thada* knockout allele were 5’-CAGATGGGATACAGAGGCTAGGCG-3’ (forward) and 5’- AATGACTGGAGATAATTGCTCATGCC-3’ (reverse), yielding a 790 bp fragment. PCR primers for wild-type allele were 5’-CAGATGGGATACAGAGGCTAGGCG-3’ (forward) and 5’- CGCACTCTTTCCCACCTGTCTTC-3’ (reverse), yielding a 517 bp fragment. *Thada*-heterozygous mice were intercrossed to generate *Thada*-knockout mice, and wild-type littermates from the same breeding pair were used as controls.

To generate *Thada*-*floxed* mice, targeting vector with the exon 31 of mouse *Thada* gene flanked by LoxP sites was engineered and electroporated into ES cells, which were then microinjected into the fertilized eggs from C57BL/6 mice. Genotypes of floxed mice were determined by PCR using the following primers: 5’- CAATGACTGTAGGTGCTGG-3’ (forward) and 5’- ACTATGTCCTTTAGTAAACTGG-3’ (reverse). To obtain β-cell-specific *Thada* knockout (β*Thada*-KO) mice, *Thada* ^*fl/fl*^ mice were crossed with *Ins1-Cre-Dsred* mice, which were kindly provided by Prof. Yanan Cao (Ruijin hospital, Shanghai Jiao Tong University School of Medicine). The Cre recombinase was expressed under the control of mouse *Ins1* promoter without *hGH* minigene and was restricted to β-cells without leakage in brain or other tissues^53^. PCR primers for *Ins1-Cre* were 5’-TAAAGCTGGTGGGCATCCAG-3’ (forward) and 5’-TCCGGTTATTCAACTTGCAC-3’ (reverse). As we found similar glucose tolerance between *Thada*^*fl/fl*^ mice and *Ins1-Cre-Dsred* mice, β*Thada*-KO (*Thada*^*fl/fl*^*,Ins1-Cre*) mice were used for experiments and their *Thada*^*fl/fl*^ littermates were used as wild-type controls.

All mice were maintained on a C57BL/6 background except *db/db* mice, and group housed on a 12 h light/dark cycle at 22-25 °C with free access to food (chow diet, 12.95% kcal fat, Beijing KEAO XIELI FEED Co., China) and water, unless indicated otherwise. Both male and female sexes were considered in the study design and analyses. Animals were monitored weekly and humane endpoints for CO_2_ euthanasia include dehydration, abnormal respiration, significant weight loss (›20% of their body weight), lethargy with ruffled fur, hunched posture, or reduced motility (inability to reach food and water). All procedures were performed in accordance with the approval of the Animal Ethics Committee of the School of Medicine, Shandong University (SDULCLL2021-2-18).

### Mouse models

Male *db/db* (C57BLKS-*Lepr*^*-/-*^) mice and their wild-type littermates at various weeks of age were purchased from Gem Pharmatech (Jiangsu, China). WT and *Thada*-KO mice at 8-week-old were switched to high-fat high-sucrose diet containing 45% kcal fat and 35% kcal carbohydrate (including 17% kcal sucrose, Research Diets, New Brunswick, NJ, USA, D12451) for up to 12 weeks. To generate high-fat diet/STZ diabetes model, WT and *Thada*-KO mice were fed a high-fat diet (60% kcal fat, Research Diets, New Brunswick, NJ, USA, D12492) starting at 4 weeks of age. After 4 weeks, mice were injected intraperitoneally with a single dose of STZ (Sigma) at 100 mg/kg (freshly dissolved in sodium citrate buffer, pH 4.5) after a 16 h fasting. Mice were maintained on HFD until the end of the study. For alnustone administration, 4-week-old C57BL/6 J were given high-fat diet (60% kcal fat, Research Diets, D12492) for a total period of 12 weeks to generate DIO mice. DIO mice were randomly divided into 2 groups and intraperitoneally injected with vehicle or alnustone (10 mg/kg) once daily for 7 days. They were maintained the high-fat diet throughout the experiment.

### Human study

Paraffin sections of pancreas far from the margin of pancreatectomy were collected from cases with partial pancreatectomy performed in the Department of Hepatobiliary Surgery in Shandong Provincial Hospital. Non-diabetes and type 2 diabetes mellitus were classified according to American Diabetes Association criteria^54^. Those who had been reported as having a malignant tumor were excluded. Then 4 cases of type 2 diabetes patients with clear diabetes history and 4 age- and BMI-matched non-diabetic participants were included in this study. Fasting blood glucose was measured within 1 week before the operation and informed consent was obtained. The detailed clinical characteristics for each participant were listed in Supplementary Data 1. The authors affirm that human research participants provided written informed consent to participate in the study and provided written informed consent for publication of the identifiable medical information included in this paper. This study was approved by the Biomedical Research Ethic Committee of Shandong Provincial Hospital and was in accordance with the principles of the Declaration of Helsinki.

### Cell culture and treatment

MIN6 and INS-1 beta-cell lines were generous gifts from Prof. Xiao Wang (Ruijin hospital, Shanghai Jiao Tong University School of Medicine). HeLa cell was purchased from National Collection of Authenticated Cell Cultures (Catalog: TCHu187). MIN6 beta-cell line (passage 18-30) was cultured in DMEM medium with 25 mM glucose, 10% fetal bovine serum (FBS), 55 μM β-mercaptoethanol, 100 U/ml penicillin and 100 μg/ml streptomycin. INS-1 beta-cell line (passage 22-35) was cultured in RPMI-1640 medium with 11.1 mM glucose, 10% FBS, 100 U/ml penicillin and 100 μg/ml streptomycin. To study the long-term effects of high glucose, INS-1 cells were cultured with RPMI-1640 medium supplemented with either 3.3 or 25 mM glucose and 0.25% bovine serum albumin (BSA) for 48 h. To study the effects of fatty acids, stock solution of palmitate conjugated to fatty acid-free BSA (5 mM palmitate and 10% BSA, Sigma) was prepared by adding 100 mM palmitate to 10.5% BSA at a 1:19 ratio. The palmitate stock solution was then diluted by pre-warmed DMEM medium to obtain the final concentration of 0.5 mM. To prepare a 2:1 mixture of oleate and palmitate, sodium palmitate was dissolved first, followed by the addition of sodium oleate (Sigma) to a final concentration of 0.5 mM. Ionomycin, CDN1163, S107 and tunicamycin were from Med Chem Express (NJ, USA). Thapsigargin was from Cell Signaling Technology (Danvers, MA, USA). Z-VAD and Z-IETD were from Selleck Chemicals (Houston, TX, USA).

### Generation of *Thada*-activated MIN6 beta-cell line

Stable MIN6 beta-cell line with endogenous *Thada*-activation was generated by CRISPR/dCas9-SAM system from Genechem (Shanghai, China). MIN6 cells were first infected with lentivirus encoding dCas9-VP64 at a multiplicity of infection (MOI) of 10. After 3 days, cells were subcultured and selected with 10 μg/ml puromycin. After 2 weeks selection, cells were infected with another lentivirus encoding sgRNA-MS2-P65-HSF1 at a MOI of 10. After 3 days, cells were subcultured and further selected with 1 mg/ml G418 for 2 weeks. The sgRNA sequence targeting mouse *Thada* gene promoter was 5’-ATCAAGAACTGTTTAGTCGC-3’. Successful activation of THADA was determined by mRNA and protein expression. Cells infected with dCas9 and non-targeting sgRNA lentiviruses were used as controls.

### Generation of *Thada*-knockout MIN6 beta-cell line

CRISPR/Cas9 system was used to generate *Thada*-knockout β-cell line. MIN6 cells were infected with lentivirus that was constructed with Cas9 and sgRNA directing at the mouse *Thada* gene (Genechem, Shanghai, China). Following infection for 3 days, cells were selected with 10 μg/ml puromycin for an additional 10 days. Selected cells were then plated at a limiting dilution. Newly isolated monoclones were expanded and screened for THADA protein expression, then monoclone-#1 was chosen for further experiments. The sgRNA targeting sequence was 5’-TTATCGACCTTCTCCAAGAG-3’.

### Isolation of pancreatic islets

Mouse pancreatic islets were isolated from WT or *Thada*-KO mice at the indicated weeks of age by collagenase digestion and dense-gradient centrifugation. Briefly, a type XI collagenase solution (Sigma) was injected through the common bile duct. The perfused pancreas was dissected and incubated at 37 °C for 17 min. Then intact islets and exocrine cells were separated via centrifugation over Histopaque-1077 (Sigma) and manually picked under the microscope. Islets were cultured in RPMI-1640 medium with 10% FBS, 100 U/ml penicillin and 100 μg/ml streptomycin. The glucolipotoxicity-inducing media was RPMI-1640 medium supplemented with 16.7 mM glucose and 0.5 mM palmitate conjugated to BSA.

### Metabolic studies

IPGTTs were performed on overnight-fasted mice by intraperitoneal injection of glucose with 2 g/kg body weight (1 g/kg dose for HFD/STZ mice). ITTs were performed on 6 h-fasted mice by intraperitoneal injection of human insulin at a dose of 0.75 U/kg for chow-diet mice and 1 U/kg for HFHS-fed mice. Blood glucose levels were measured with a portable glucometer (Accu-Chek, Roche) at 0, 15, 30, 60, and 120 min after glucose injection. For insulin release test, blood sample were taken from the tail vein at 0, 15, and 30 min after 2 g/kg glucose injection, then serum insulin levels were determined using Mouse Ultrasensitive Insulin ELISA kits (ALPCO, Salem, NH, USA).

### Immunostaining analysis

Pancreases were dissected, fixed, and processed, as described before^55^. For immunofluorescence, pancreatic sections or cells grown on culture slides were stained with the following antibodies: guinea pig anti-insulin (1:5, DAKO, #IR00261-2), rabbit anti-THADA (1:400, Sigma, #HPA035192), mouse anti-glucagon (1:200, Abcam, #ab10988), rabbit anti-Ki67 (1:500, Cell Signaling Technology, #9129), mouse anti-SERCA2 (1:500, Abcam, #ab2861), mouse anti-RyR (1:200, Invitrogen, #MA3-916), mouse anti-DR5 (1:200, Santa Cruz, sc-166624), mouse anti-FADD (1:200, Millipore, #05-486), rabbit anti-cleaved caspase-8 (1:400, Cell Signaling Technology, #8592), rabbit anti-cleaved caspase-3 (1:400, Cell Signaling Technology, #9664), mouse anti-ATP5A (1:500, Abcam, #ab14748). CellLight Mitochondria-GFP (ThermoFisher) was used to label mitochondria following the manufacturer’s instructions. Detection was performed using Alexa Fluor 488 and 594 (Invitrogen). All sections were counterstained with DAPI (Vectashield mounting medium with DAPI, Vector Laboratories, USA). Images were captured by a confocal microscope (Andor Dragonfly). β-cell proliferation was calculated using the percentage of Ki67^+^Insulin^+^ cells and at least 2500 β-cells per animal were counted. β-cell apoptosis was measured using the In Situ Cell Death Detection Kit (Roche) following the manufacturer’s instructions. The percentage of Tunel^+^Insulin^+^ cells was calculated and at least 3000 β-cells per animal were counted. For cleaved caspase-8 and cleaved caspase-3 quantifications, at least 2500 β-cells per animal were counted. Measurements of fluorescence intensities in insulin-positive area as well as co-localization analyses were performed by ImageJ software (National Institutes of Health). To measure β-cell mass, at least 10 evenly 200 μm apart sections throughout the entire pancreas were picked to perform immunochemistry staining of insulin with a DAB Peroxidase Substrate Kit (Vector Laboratories, USA). Sections were then counterstained with eosin. Digital images of whole pancreas were captured by a Olympus VS120 microscope (Tokyo, Japan). The insulin-positive area and total pancreatic area were measured using ImageJ software. β-cell mass/α-cell mass was calculated as the ratio of insulin-positive area (glucagon-positive area) to total pancreatic area multiplied by pancreatic weight.

### Insulin secretion assay

Isolated islets from WT or *Thada*-KO mice were cultured with RPMI-1640 medium containing 0.25% BSA. To stimulate insulin secretion, islets were pre-incubated in Krebs-Ringer bicarbonate (KRB) buffer containing 3.3 mM glucose for 30 min. Batches of ten size-matched islets were then incubated in KRB buffer containing 3.3, 8.3, 11.1, or 16.7 mM glucose as indicated for 1 h at 37 °C. The supernatants were collected for the measurement of insulin secretion. Islets were then extracted with 0.18 N HCl in 70% ethanol to determine total insulin content. Insulin levels were measured using a Mouse Insulin ELISA kit (ALPCO, Salem, NH, USA) and insulin secretion was normalized as percentage of total insulin content.

MIN6 cells were seeded in 24-well plates and cultured to confluence. Cells were pre-incubated in KRB buffer containing 2.8 mM glucose for 30 min. After pre-incubation, cells were stimulated for 1 h in KRB buffer containing various concentrations of glucose as indicated or 2.8 mM glucose supplemented with 2.5 mM tolbutamide (Sigma) or 35 mM KCl. Secretion buffer was sampled and assayed using the Mouse Insulin ELISA kit (ALPCO, Salem, NH, USA). Cells were lysed in ice-cold RIPA buffer and lysates were assayed for protein concentration using the BCA Protein Assay Kit (ThermoFisher Scientific, MA, USA). Data are presented as insulin secreted per total cellular protein.

### Pancreatic insulin content and ATP measurement

Whole pancreatic tissues were dissected and homogenized in acid-ethanol solution. After an overnight incubation at 4 °C, the extracts were centrifuged and supernatants collected. Insulin was measured using a mouse insulin ELISA kit (ALPCO) and was normalized over protein concentration as determined by BCA assay. For ATP measurement, batches of ten islets each condition were lysed following the procedure of GSIS. Then ATP levels were measured using an Enhanced ATP Assay Kit (Beyotime) by a luminometer (Molecular Devices, CA, USA).

### Electron microscopy

The pancreas was fixed, dehydrated, sectioned and imaged. The subtype, density and diameter of insulin vesicles were determined by counting images captured at 2000× magnification. Representative images were captured at 5000× magnification. Each analysis was based on at least eight images per mice.

### Cytosolic Ca^2+^ measurement

Cytosolic Ca^2+^ level in MIN6 beta-cell line was determined using the ratiometric Ca^2+^ indicator Fura-2 AM (Sigma). Cells seeded on the glass-bottom culture dish were loaded with 2 μM Fura-2 AM combined with Pluronic F-127 (Invitrogen, CA, USA) in KRB buffer containing 2.8 mM glucose at 37 °C for 30 min. After rinsing, cells were placed under a fluorescence microscope (Nikon Eclispse Ts2R, Tokyo, Japan) and alternatively excited at 340 and 380 nm. Stimuli were applied with the bathing solution. Changes in the 340/380 nm fluorescence emission ratio after the addition of high glucose (25 mM) or KCl (30 mM) were analyzed over time in individual cells using MetaFluor imaging software. Peak changes in the fluorescence ratio were measured to compare response profiles. Change in Fura-2 fluorescence signal (ΔF) was normalized to baseline fluorescence (F0). The maximum increases in cytosolic Ca^2+^ are presented as ΔF /F0. To measure ER Ca^2+^ store, cells were incubated in KRB buffer without Ca^2+^ and supplemented with 1 mM EGTA after Fura-2 loading. Then 10 μM ionomycin or 1 μM thapsigargin was added to release ER Ca^2+^ storage. Changes in fluorescence ratio were analyzed by MetaFluor software. The maximum increases in cytosolic Ca^2+^ are presented as ΔF /F0.

For Ca^2+^ measurements in islets, primary isolated islets were dispersed with trypsin-EDTA and then seeded into poly-L-lysine-coated glass-bottom plates. After attachment, all islet cells were loaded with fluo-4 Ca^2+^ indicator (ThermoFisher Scientific, MA, USA) combined with Pluronic F-127 in KRB buffer at the indicated glucose concentration for 50 minutes. Then islet cells were washed in indicator-free KRB buffer and incubated for a further 30 minutes. Fluorescence was measured using an Enspire fluorescent spectrophotometer system (PerkinElmer) before and after cells were stimulated with 25 mM glucose, 35 mM KCl, 10 μM ionomycin, or 10 μM IP3 (sigma) as indicated. For ionomycin treatment, cells were incubated in KRB buffer without Ca^2+^ and supplemented with 1 mM EGTA after fluo-4 loading. The mean fluorescence intensity per well was calculated and maximum increases in [Ca^2+^]_i_ are presented as ΔF /F0.

### Cell viability and apoptosis assay

Cell viability was determined by CCK-8 assay (Beyotime) according to the manufacturer’s instructions. An Apoptosis Detection Kit (Multi Sciences, Hangzhou, China) was used to detect cell apoptosis. Briefly, MIN6 beta-cell lines treated with the indicated reagents were collected and washed with binding buffer. Cells were then stained with Annexin V and 7-AAD and analyzed by BD LSRFortessa flow cytometer. Cell apoptosis was calculated as the percentage of Annexin V-positive early and late apoptotic cells. Caspase-Glo 8 Assay (Promega) and Caspase-Glo 3/7 Assay (Promega) were used to measure caspase-8 and caspase-3/7 activities following the manufacturer’s instructions. Briefly, cells seeded in 96-well plate were incubated with the Caspase-Glo Reagent for 30 min. Then luminescence of each sample was measured in a plate-reading luminometer (PerkinElmer).

### Western blot and immunoprecipitation

Cells or islets were collected and lysed by RIPA buffer containing protease and phosphatase inhibitors (Beyotime). Protein concentration was determined using the BCA Protein Assay Kit (ThermoFisher Scientific, Waltham, MA, USA). 30 μg of total protein lysates were subjected to 4%-12% NuPAGE gels (Invitrogen) and transferred to PVDF membranes (Millipore, MA, USA). After blocking with 5% non-fat milk for 1 h, membranes were incubated with the following primary antibodies at 1:1000 dilution overnight at 4 °C: anti-THADA (Sigma, #HPA035192), anti-SERCA2 (Abcam, #ab2861), anti-RyR (Invitrogen, #MA3-916), anti-cleaved caspase-8 (Cell Signaling Technology, #8592), anti-caspase-8 (Cell Signaling Technology, #4927), anti-cleaved caspase-3 (Cell Signaling Technology, #9664), anti-ATF4 (Cell Signaling Technology, #11815), anti-CHOP (Cell Signaling Technology, #2895), anti-FADD (Millipore, #05-486), anti-DR5 (Abcam, #ab8416), anti-phospho-IP3R (Cell Signaling Technology, #3760), anti-IP3R (Santa Cruz, #sc377518), anti-HSP90 (Cell Signaling Technology #4874), anti-GAPDH (Proteintech #60004-1-Ig), anti-β-Actin (Proteintech #60008-1-Ig). Membranes were exposed to secondary antibodies for 1 h at room temperature and developed using Immobilon Western HRP Substrate (Millipore, MA, USA) with a Bio-Rad imaging system. Immunoprecipitation was performed by incubating protein lysates with the indicated antibodies for 2 h and then with Protein A/G Plus agarose beads (Santa Cruz) overnight at 4 °C. The binding complexes were washed with lysis buffer and eluted with loading buffer. Standard western blotting was then followed using the antibodies indicated above. The full scan blots are provided in the Source Data file.

### Proximity ligation assay

The proximity ligation assay (PLA) was performed in MIN6 beta-cell line grown on Nunc LAB-Tek chamber slide (ThermoFisher Scientific) according to the manufacturer’s protocol (Sigma, DuoLink). Combinations of primary rabbit and mouse antibodies were incubated overnight at 4 °C. THADA was detected with anti-rabbit PLUS PLA probe and SERCA2/RyR was detected with anti-mouse MINUS PLA probe. Nuclei were stained with DAPI. PLA signals were detected with a confocal microscope (Andor Dragonfly) as discrete spots in red.

### RNA Sequencing

Total RNA was extracted from control and *Thada*-activated MIN6 beta-cell lines (*n* = 3). The sequencing library was generated using an Agilent 2100 Bioanalyzer (Agilent Technologies, Santa Clara, CA, USA). After fragment screening, library building and PCR product purification, the samples were sequenced on the BGISEQ-500 platform (Wuhan, China). The differentially expressed genes were identified with a q-value <0.05 and a fold-change >1.5 between the two groups.

### RNA isolation and quantitative PCR

Total RNA from islets or cells were extracted using RNeasy Mini Kit (Qiagen, Hiden, Germany) and reverse transcribed using the Prime Script RT Kit with gDNA Eraser (Takara, Shiga, Japan). Real-time quantitative PCR was performed using SYBR Premix Ex Taq Kit (Takara, Shiga, Japan) on a Light Cycler 480 System (Roche, Basel, Switzerland). Results were normalized to *β-Actin* mRNA levels. The primer sequences are provided in Supplementary Data 2.

### High-content screen

To perform the high-content screen, *Thada*-activated MIN6 cells were plated onto 96-well plates at 50,000 cells per well. Cells were treated at 10 μM with a compound library containing FDA-approved drugs as well as traditional Chinese medicine monomers (purchased from Topscience, Shanghai, China) and DMSO treatment was used as a negative control. After 24 hours, these cells were loaded with fluo-4 Ca^2+^ indicator (ThermoFisher Scientific, MA, USA) at 2.8 mM glucose. The plates were then imaged on the Opera Phenix high-content screening system (PerkinElmer) using 20× objective and five image fields were collected from each well. After further stimulated with 25 mM glucose, the cells were immediately imaged for another measurement. For each measurement, laser power and exposure times were adjusted to the linear detection range. Images were analyzed using Harmony 4.9 software (PerkinElmer) and the mean fluorescence intensity per well was calculated. Compounds increasing 25 mM glucose-stimulated [Ca^2+^]_i_ in *Thada*-activated cells compared to DMSO treated wells were selected as primary hits. Z score was calculated as (25 mM glucose-stimulated [Ca^2+^]_i_ of compound treated condition – mean [Ca^2+^]_i_)/ STDEV of the [Ca^2+^]_i_.

### Statistical analysis

The results are expressed as mean ± SEM for the indicated number of observations. Data were analyzed using two-tailed Student’s *t* test for two groups or one-way ANOVA for multiple groups. Differences with *p* < 0.05 were considered statistically significant.

### Reporting summary

Further information on research design is available in the Nature Portfolio Reporting Summary linked to this article.

## Supplementary information

Supplementary Information

Description of Additional Supplementary Files

Supplementary Data 1

Supplementary Data 2

Reporting Summary

## Data Availability

The RNA sequencing data generated in this study have been deposited in the Gene Expression Omnibus database under accession code GSE173267. All other data generated or analyzed during this study are included in this published article (and its supplementary information files). Source data are provided with this paper.

## Source data

Source Data

## References

[1] Ashcroft FM, Rorsman P (2012). Diabetes mellitus and the beta cell: the last ten years. Cell..

[2] Krentz N, Gloyn A (2020). Insights into pancreatic islet cell dysfunction from type 2 diabetes mellitus genetics. Nat. Rev. Endocrinol..

[3] Holman RR, Clark A, Rorsman P (2020). β-cell secretory dysfunction: a key cause of type 2 diabetes. Lancet Diabetes Endocrinol..

[4] Weir GC, Gaglia J, Bonner-Weir S (2020). Inadequate β-cell mass is essential for the pathogenesis of type 2 diabetes. Lancet Diabetes Endocrinol..

[5] Rippe V (2003). Identification of a gene rearranged by 2p21 aberrations in thyroid adenomas. Oncogene.

[6] Zeggini E (2008). Meta-analysis of genome-wide association data and large-scale replication identifies additional susceptibility loci for type 2 diabetes. Nat. Genet..

[7] Mahajan A (2018). Fine-mapping type 2 diabetes loci to single-variant resolution using high-density imputation and islet-specific epigenome maps. Nat. Genet..

[8] Morris A (2012). Large-scale association analysis provides insights into the genetic architecture and pathophysiology of type 2 diabetes. Nat. Genet..

[9] Prasad R (2016). Excess maternal transmission of variants in the THADA gene to offspring with type 2 diabetes. Diabetologia.

[10] Simonis-Bik A (2010). Gene variants in the novel type 2 diabetes loci CDC123/CAMK1D, THADA, ADAMTS9, BCL11A, and MTNR1B affect different aspects of pancreatic beta-cell function. Diabetes.

[11] Stancáková A (2009). Association of 18 confirmed susceptibility loci for type 2 diabetes with indices of insulin release, proinsulin conversion, and insulin sensitivity in 5,327 nondiabetic Finnish men. Diabetes.

[12] Hu C (2009). PPARG, KCNJ11, CDKAL1, CDKN2A-CDKN2B, IDE-KIF11-HHEX, IGF2BP2 and SLC30A8 are associated with type 2 diabetes in a Chinese population. PLoS ONE.

[13] Chen Z (2011). Genome-wide association study identifies susceptibility loci for polycystic ovary syndrome on chromosome 2p16.3, 2p21 and 9q33.3. Nat. Genet..

[14] Cardona A (2014). Genome-wide analysis of cold adaptation in indigenous Siberian populations. PLoS ONE.

[15] Moraru A (2017). THADA regulates the organismal balance between energy storage and heat production. Dev. cell.

[16] Dong, Y., Teleman, A., Jedmowski, C., Wirtz, M. & Hell, R. The Arabidopsis THADA homologue modulates TOR activity and cold acclimation. *Plant Biology (Stuttgart, Germany)*, 77-83, 10.1111/plb.12893 (2019). 10.1111/plb.12893 30098100

[17] Drieschner N (2007). A domain of the thyroid adenoma associated gene (THADA) conserved in vertebrates becomes destroyed by chromosomal rearrangements observed in thyroid adenomas. Gene.

[18] Poitout V, Robertson R (2008). Glucolipotoxicity: fuel excess and beta-cell dysfunction. Endocr. Rev..

[19] Cheng A (2013). Multiplexed activation of endogenous genes by CRISPR-on, an RNA-guided transcriptional activator system. Cell Res..

[20] Prentki M, Matschinsky FM, Madiraju SR (2013). Metabolic signaling in fuel-induced insulin secretion. Cell Metab..

[21] Sabatini P, Speckmann T, Lynn F (2019). Friend and foe: β-cell Ca signaling and the development of diabetes. Mol. Metab..

[22] Tamarina N, Kuznetsov A, Rhodes C, Bindokas V, Philipson L (2005). Inositol (1,4,5)-trisphosphate dynamics and intracellular calcium oscillations in pancreatic beta-cells. Diabetes.

[23] Zhang, I., Raghavan, M. & Satin, L. The endoplasmic reticulum and calcium homeostasis in pancreatic beta cells. *Endocrinology* **161**, 10.1210/endocr/bqz028 (2020). 10.1210/endocr/bqz028 PMC7028010 31796960

[24] Kono T (2012). PPAR-γ activation restores pancreatic islet SERCA2 levels and prevents β-cell dysfunction under conditions of hyperglycemic and cytokine stress. Mol. Endocrinol..

[25] Brillantes A (1994). Stabilization of calcium release channel (ryanodine receptor) function by FK506-binding protein. Cell.

[26] Zalk R (2015). Structure of a mammalian ryanodine receptor. Nature.

[27] Santulli G (2015). Calcium release channel RyR2 regulates insulin release and glucose homeostasis. J. Clin. Investig..

[28] Kang S (2016). Small molecular allosteric activator of the sarco/endoplasmic reticulum Ca2+-ATPase (SERCA) attenuates diabetes and metabolic disorders. J. Biol. Chem..

[29] Oyadomari S (2001). Nitric oxide-induced apoptosis in pancreatic beta cells is mediated by the endoplasmic reticulum stress pathway. Proc. Natl. Acad. Sci. USA.

[30] Hetz C (2012). The unfolded protein response: controlling cell fate decisions under ER stress and beyond. Nat. Rev. Mol. Cell Biol..

[31] Lu M (2014). Opposing unfolded-protein-response signals converge on death receptor 5 to control apoptosis. Science.

[32] Wilson N, Dixit V, Ashkenazi A (2009). Death receptor signal transducers: nodes of coordination in immune signaling networks. Nat. Immunol..

[33] Mu J (2006). Chronic inhibition of dipeptidyl peptidase-4 with a sitagliptin analog preserves pancreatic beta-cell mass and function in a rodent model of type 2 diabetes. Diabetes.

[34] Yang K, Chan C (2017). Proposed mechanisms of the effects of proanthocyanidins on glucose homeostasis. Nutr. Rev..

[35] Claeson P (1993). Three non-phenolic diarylheptanoids with anti-inflammatory activity from Curcuma xanthorrhiza. Planta Med..

[36] Osman W (2020). Genetics of type 2 diabetes and coronary artery disease and their associations with twelve cardiometabolic traits in the United Arab Emirates population. Gene.

[37] Diamanti-Kandarakis E, Dunaif A (2012). Insulin resistance and the polycystic ovary syndrome revisited: an update on mechanisms and implications. Endocr. Rev..

[38] Dadachanji, R., Sawant, D., Patil, A. & Mukherjee, S. THADAReplication study of rs13429458 variant with PCOS susceptibility and its related traits in Indian women. *Gynecol. Endocrinol* 1–5, 10.1080/09513590.2021.1906854 (2021). 10.1080/09513590.2021.1906854 33779462

[39] Tian Y (2020). THADA, INSR, TOX3PCOS-GWAS susceptibility variants in, and are associated with metabolic syndrome or insulin resistance in women with PCOS. Front. Endocrinol..

[40] Gambineri A (2012). Polycystic ovary syndrome is a risk factor for type 2 diabetes: results from a long-term prospective study. Diabetes.

[41] Thomsen S (2016). Systematic functional characterization of candidate causal genes for type 2 diabetes risk variants. Diabetes.

[42] Torres J (2020). A multi-omic integrative scheme characterizes tissues of action at loci associated with type 2 diabetes. Am. J. Hum. Genet..

[43] Luciani D (2009). Roles of IP3R and RyR Ca2+ channels in endoplasmic reticulum stress and beta-cell death. Diabetes.

[44] Thore S, Dyachok O, Tengholm A (2004). Oscillations of phospholipase C activity triggered by depolarization and Ca2+ influx in insulin-secreting cells. J. Biol. Chem..

[45] Mitchell K, Lai F, Rutter G (2003). Ryanodine receptor type I and nicotinic acid adenine dinucleotide phosphate receptors mediate Ca2+ release from insulin-containing vesicles in living pancreatic beta-cells (MIN6). J. Biol. Chem..

[46] Sladek R (2007). A genome-wide association study identifies novel risk loci for type 2 diabetes. Nature.

[47] Tong X (2016). SERCA2 deficiency impairs pancreatic β-cell function in response to diet-induced obesity. Diabetes.

[48] Ghosh, R., Colon-Negron, K. & Papa, F. Endoplasmic reticulum stress, degeneration of pancreatic islet β-cells, and therapeutic modulation of the unfolded protein response in diabetes. *Mol. Metab.* S60-S68, 10.1016/j.molmet.2019.06.012 (2019). 10.1016/j.molmet.2019.06.012 PMC6768499 31500832

[49] Lam M, Lawrence D, Ashkenazi A, Walter P (2018). Confirming a critical role for death receptor 5 and caspase-8 in apoptosis induction by endoplasmic reticulum stress. Cell Death Differ..

[50] Lam, M., Marsters, S., Ashkenazi, A. & Walter, P. Misfolded proteins bind and activate death receptor 5 to trigger apoptosis during unresolved endoplasmic reticulum stress. *eLife* **9**, 10.7554/eLife.52291 (2020). 10.7554/eLife.52291 PMC7041945 31904339

[51] Eizirik D, Pasquali L, Cnop M (2020). Pancreatic β-cells in type 1 and type 2 diabetes mellitus: different pathways to failure. Nat. Rev. Endocrinol..

[52] Christodoulou M (2019). Blood-based analysis of type-2 diabetes mellitus susceptibility genes identifies specific transcript variants with deregulated expression and association with disease risk. Sci. Rep..

[53] Cheng Y (2015). Generation and characterization of transgenic mice expressing mouse ins1 promoter for pancreatic beta-cell-specific gene overexpression and knockout. Endocrinology.

[54] Standards of Medical Care in Diabetes-20212. (2021). Classification and diagnosis of diabetes. Diabetes Care.

[55] Zhang Y (2020). Protein acetylation derepresses serotonin synthesis to potentiate pancreatic beta-cell function through HDAC1-PKA-Tph1 signaling. Theranostics.

