# Supplementary material for: THADA inhibition in mice protects against type 2 diabetes mellitus by improving pancreatic β-cell function and preserving β-cell mass
Source: Nat Commun. 2023 Feb 23;14:1020. doi: 10.1038/s41467-023-36680-0 (PMC9950491; doi:10.1038/s41467-023-36680-0)
Supplement: Supplementary file 1 — Supplementary Information [file 41467_2023_36680_MOESM1_ESM.pdf]

Supplementary Information

THADA Inhibition in mice Protects against Type 2 Diabetes Mellitus by Improving Pancreatic β-cell Function and Preserving β-cell Mass

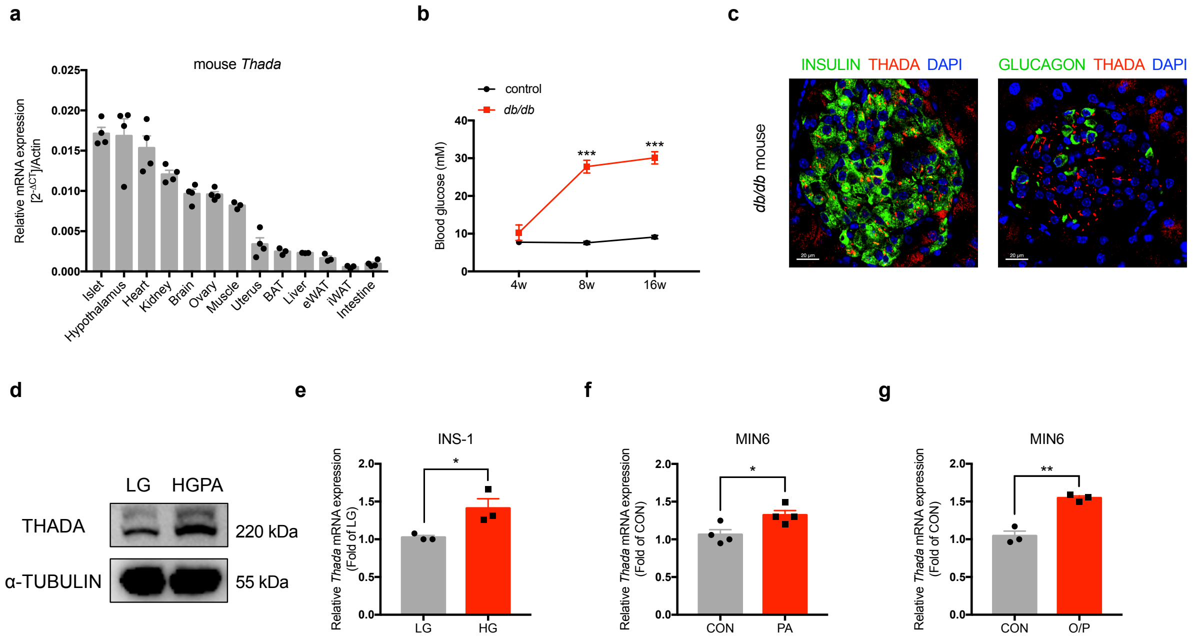

Figure S1. *Thada* expression is increased after glucolipotoxicity in β-cells.

(**a**) The mRNA expression levels of *Thada* in mouse islet (n=4), hypothalamus (n=4), heart (n=4), kidney (n=4), brain (n=4), ovary (n=4), muscle(n=3), uterus (n=4), brown adipose tissue (BAT, n=3), liver (n=3), epididymal white adipose tissue (eWAT, n=3), subcutaneous inguinal white adipose tissue (iWAT, n=3), and intestine (n=4) were determined. (**b**) Blood glucose levels of *db/db* mice and wild-type control littermates at 4, 8 and 16 weeks of age (n=3). (**c**) Representative pancreatic sections from 8-week-old *db/db* mice staining for insulin (green) and THADA (red), or glucagon (green) and THADA (red). Scale bars, 20 μm. (**d**) THADA protein levels in mouse islets incubated with 3.3 mM glucose (LG) or 16.7 mM glucose combined with 0.5 mM palmitate (HGPA) for 48 h. (**e**) The mRNA expression level

of *Thada* was determined after INS-1 cells were treated with 3.3 mM (LG) or 25 mM glucose (HG) for 48 h (n=3). (**f**) The mRNA expression level of *Thada* was determined after MIN6 beta-cell lines were treated with 0.5 mM palmitate (PA) for 48 h (n=4). (**g**) The mRNA expression level of *Thada* was determined after MIN6 beta-cell lines were treated with a 2:1 mixture of oleate and palmitate (O/P, total concentration was 0.5 mM) for 48 h (n=3). The western blots and immunostainings show representative results from at least three independent experiments. Data are presented as mean ± SEM. *P<0.05, **P<0.01, ***P<0.001; two-tailed unpaired Student's *t* test. Source data are provided as a source data file.

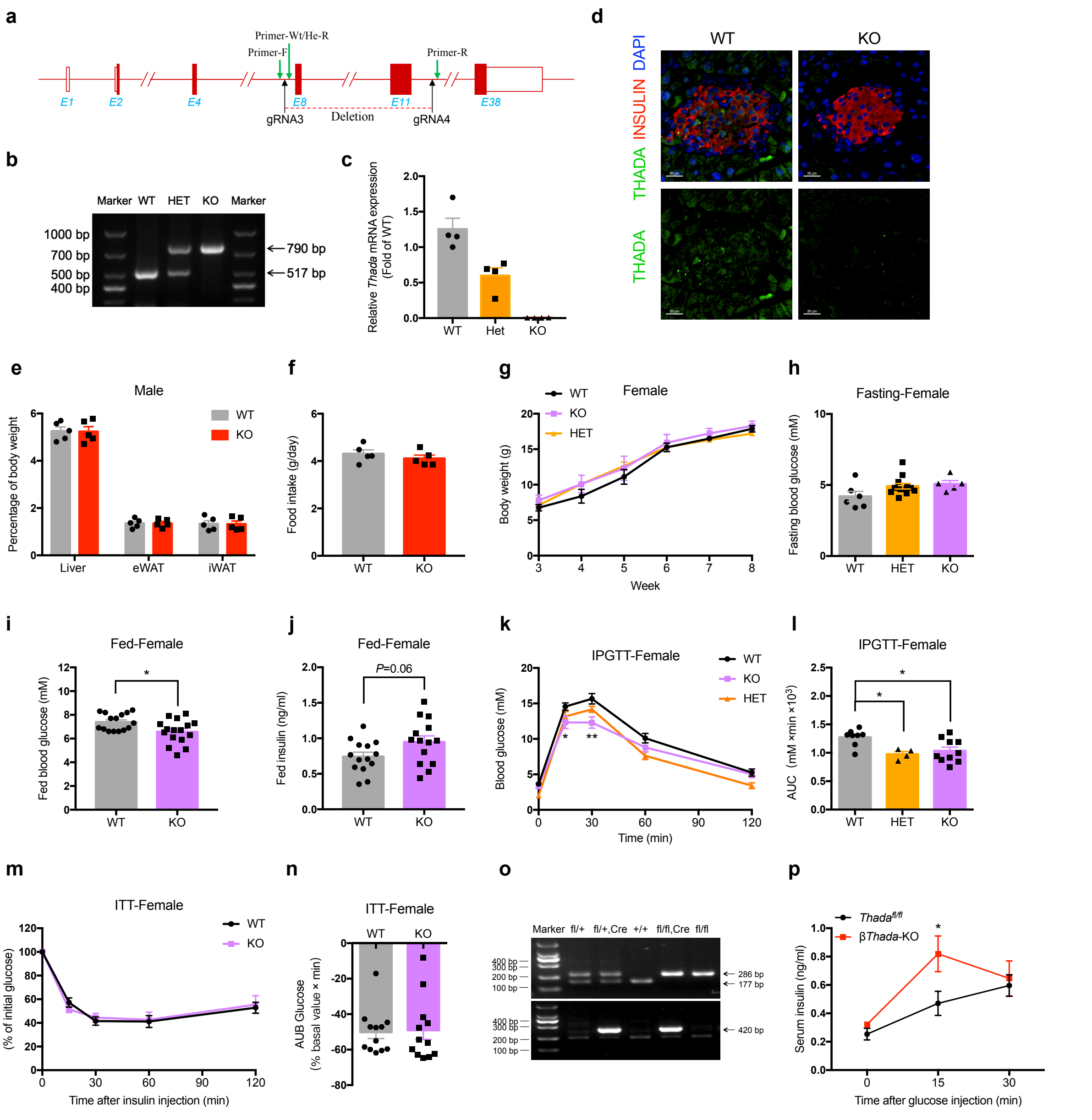

Figure S2. ***Thada*** **knockout improves glucose homeostasis without affecting insulin sensitivity in female mice.**

(**a**) Generation of *Thada*-knockout mice by CRISPR-Cas9. (**b**) Genotyping for wild-type (WT), heterozygous (HET), and *Thada*-knockout (KO) mice. The wild-type allele yielded a 517 bp fragment while knockout allele yielded a 790 bp fragment. (**c**) Detection of *Thada* mRNA expression in islets isolated from WT, HET and *Thada*-KO male mice (n=4). (**d**) Immunofluorescence staining for THADA in pancreatic sections from WT and *Thada*-KO

mice. Scale bars, 20 μm. (**e**) Weights of liver, epididymal white adipose tissue (eWAT), inguinal white adipose tissue (iWAT) normalized to body weight in WT and *Thada*-KO male mice (n=5). (**f**) The average food intake per day of WT and *Thada*-KO male mice (n=5). (**g**) Body weights of WT (n=4), HET (n=8) and *Thada*-KO female mice (n=4) at the indicated weeks of age. (**h**) Blood glucose levels of WT (n=6), HET (n=12) and *Thada*-KO female mice (n=5) that were fasted overnight. (**i**) Random-fed blood glucose levels of WT (n=15) and *Thada*-KO female mice (n=16). (**j**) Fed serum insulin levels of female mice treated as in F (n=14). (**k** and **l**) IPGTT and area under the curve (AUC) of WT (n=8), HET (n=4) and *Thada*-KO (n=10) female mice. (**m** and **n**) ITT and area under baseline (AUB) of WT and *Thada*-KO female mice (n=12). (**o**) Genotyping for $Thada^{fl/fl}$ and $Thada^{fl/fl}$,*Ins1-Cre* mouse. The floxed allele yielded a 286 bp fragment, WT allele yielded a 177 bp fragment, and *Ins1-Cre* yielded a 420 bp fragment. (**p**) Serum insulin levels at 0, 15, and 30 min after intraperitoneal glucose injection in $Thada^{fl/fl}$ and β*Thada*-KO male mice (n=5). All the experiments were performed on mice at 8-10 weeks of age. Data are presented as mean ± SEM. **P*<0.05, ***P*<0.01; two-tailed unpaired Student's *t* test. Source data are provided as a source data file.

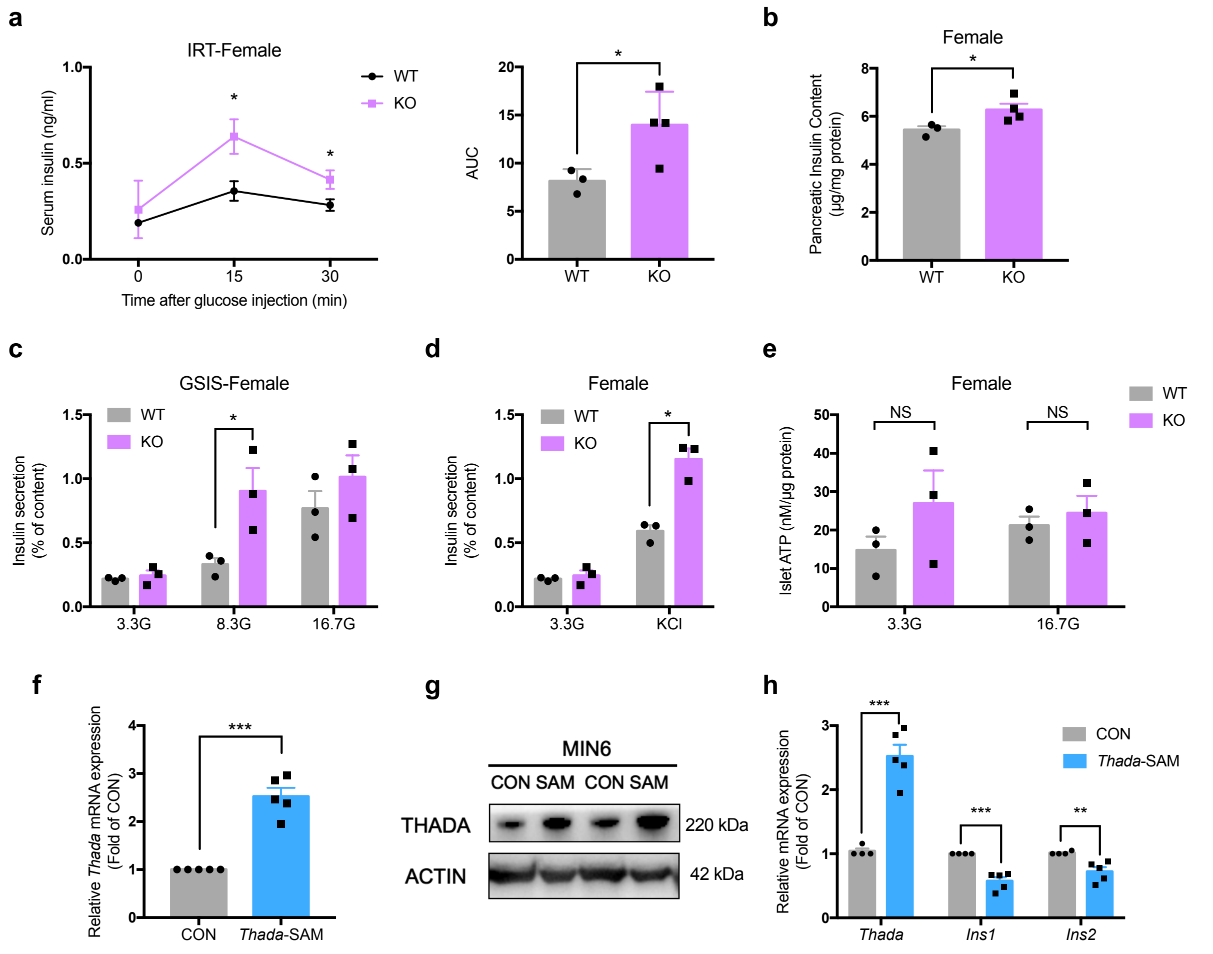

Figure S3. ***Thada*** **knockout promotes pancreatic β-cell function in female mice.**

(**a**) Serum insulin levels at 0, 15, and 30 min after intraperitoneal glucose injection and area under the curve in WT (n=3) and *Thada*-KO (n=4) mice. (**b**) Pancreatic insulin content of WT (n=3) and *Thada*-KO (n=4) mice. (**c**) Islets isolated from WT and *Thada*-KO mice were stimulated with 3.3, 8.3, and 16.7 mM glucose for 1 h and insulin secretion was assayed (n=3). (**d**) Islets isolated from WT and *Thada*-KO mice were stimulated with or without 35 mM KCl at 3.3 mM glucose for 1 h and insulin secretion was assayed (n=3). (**e**) ATP content of islets from WT and *Thada*-KO mice incubated at 3.3 or 16.7 mM glucose for 1 h (n=3). (**f and g**) The mRNA (f, n=5) and protein (g) expression levels of THADA were detected in control (CON) and *Thada*-activated (*Thada*-SAM) MIN6 beta-cell lines. (**h**) The mRNA expression

levels of *Thada*, *Ins1*, and *Ins2* were determined in control (n=4) and *Thada*-SAM (n=5) MIN6 beta-cell lines. All animal experiments were performed on female mice at 8-10 weeks of age. Data are presented as mean ± SEM. *$P<0.05$, **$P<0.01$, ***$P<0.001$; two-tailed unpaired Student's t test. Source data are provided as a source data file.

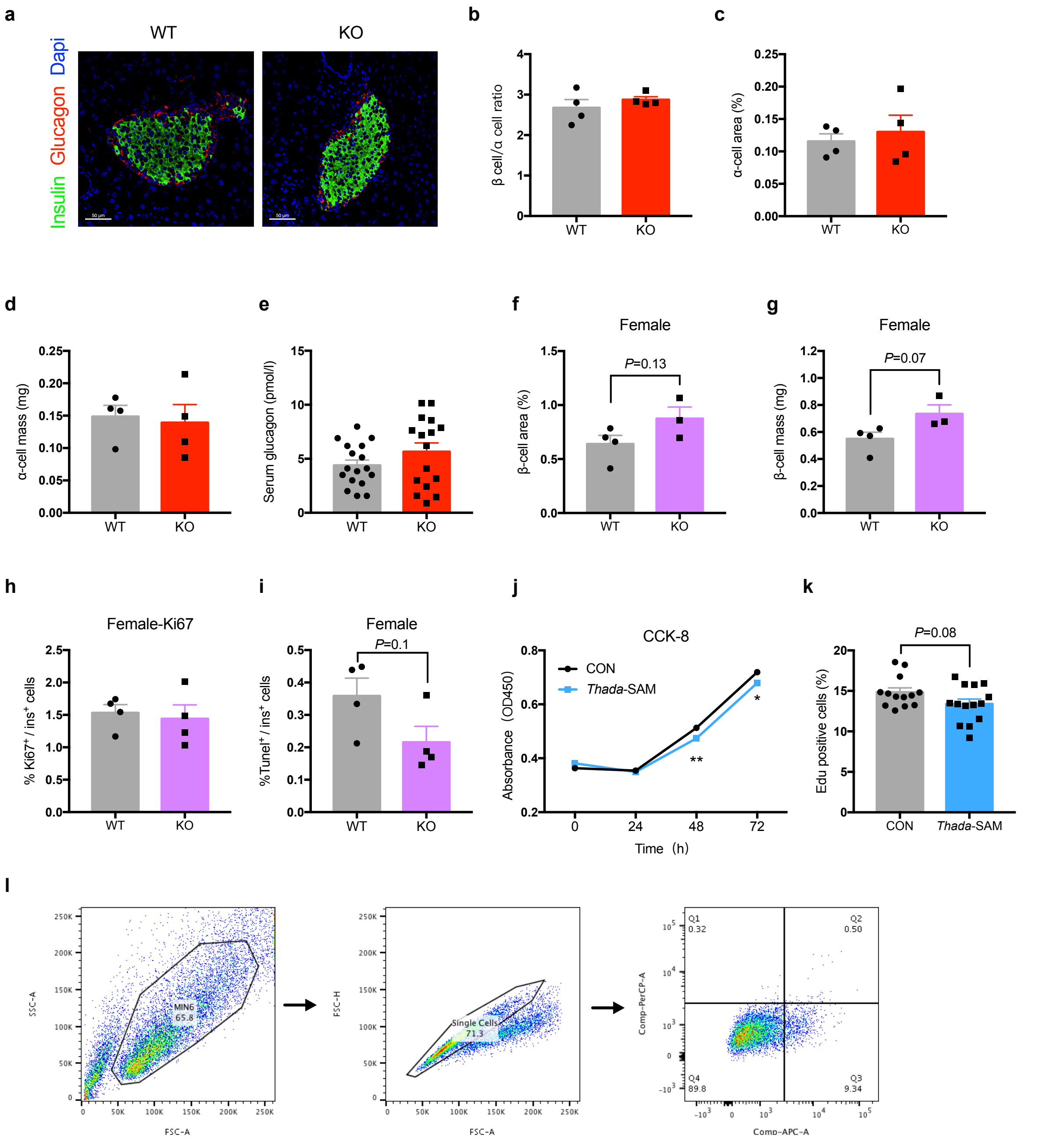

Figure S4. *Thada*-knockout mice have increased β-cell mass owing to reduced apoptosis.

(**a**) Representative pancreatic sections from WT and *Thada*-KO male mice stained for insulin (green), glucagon (red) and DAPI (blue). Scale bars, 50 μm. (**b**) The ratio of insulin-positive cells to glucagon-positive cells was calculated in WT and *Thada*-KO male mice (n=4). (**c**) Measurements of α-cell area/pancreatic area ratio in WT and *Thada*-KO male mice (n=4). (**d**) Measurements of α-cell mass in WT and *Thada*-KO male mice (n=4). (**e**) Serum glucagon levels of WT (n=17) and *Thada*-KO (n=16) male mice at fed state. (**f**) Measurements of β-cell area/pancreatic area ratio in WT (n=4) and *Thada*-KO (n=3) female mice. (**g**) Measurements of β-cell mass in WT (n=4) and *Thada*-KO (n=3) female mice. (**h**) The proliferation of β-cell from WT and *Thada*-KO female mice (n=4) was determined by quantification the percentage of $Ki67^+$ in $Insulin^+$ cells. 5611±1466 $Insulin^+$ cells were quantified for WT mice and 5415±1289 $Insulin^+$ cells were quantified for KO mice. (**i**) The apoptosis of β-cell from WT and *Thada*-KO female mice (n=4) was determined by quantification the percentage of $Tunel^+$ in $Insulin^+$ cells. 4301±1168 $Insulin^+$ cells were quantified for WT mice and 4062±579 $Insulin^+$ cells were quantified for KO mice. (**j**) CCK-8 assay of control and *Thada*-SAM β-cells at various time points (n=8 for 0 h, n=10 for 24 and 48 h, n=9 for 72 h). (**k**) The proliferation of control (n=13) and *Thada*-SAM (n=14) β-cells was determined by Edu assay and percentage of Edu-positive cells was calculated. (**l**) Gating strategy of apoptosis analysis. Starting MIN6 cell population was determined by FSC-A/SSC-A gating and single cells were gated by FSC-A/FSC-H. All animal experiments were performed on mice at 8-10 weeks of age. Data are presented as mean ± SEM. *P<0.05, **P<0.01; two-tailed unpaired Student's t test. Source data are provided as a source data file.

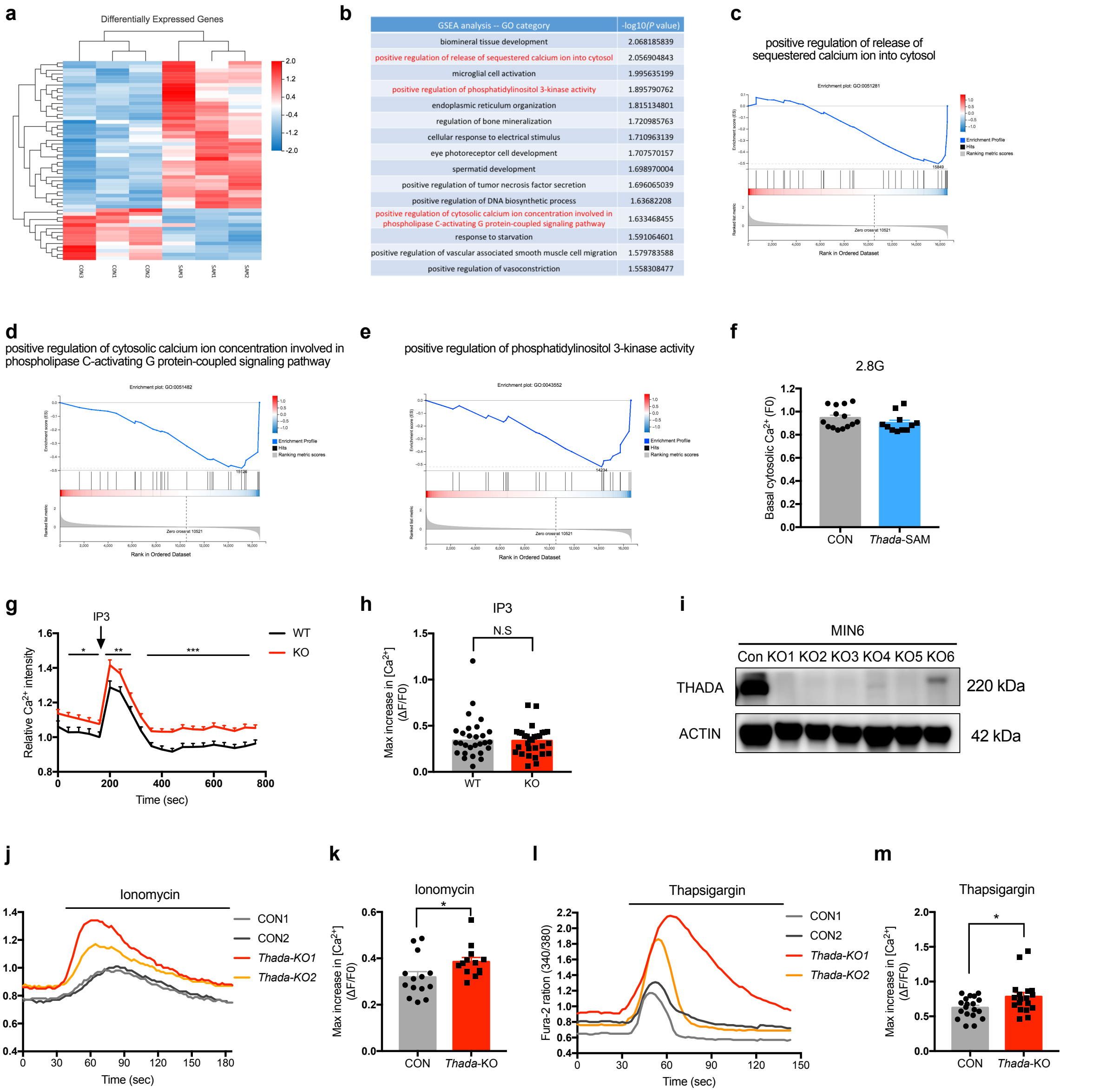

Figure S5. THADA reduces ER calcium stores in β-cells.

(**a**) Heat map shows the differentially expressed genes between control and *Thada*-activated β-cells. (**b**) Top 15 enriched GO categories by GSEA for *Thada* activation. (**c-e**) GSEA plots demonstrating genes enriched in "positive regulation of release of sequestered calcium ion into cytosol" (**c**), "positive regulation of cytosolic calcium ion concentration involved in phospholipase C-activating G protein-coupled signaling pathway" (**d**) and "positive regulation of phosphatidylinositol 3-kinase activity" (**e**). (**f**) Quantification of basal $[Ca^{2+}]_i$ under 2.8 mM glucose from control (CON) and *Thada*-activated (*Thada*-SAM) MIN6

beta-cell lines (n=14 for control and n=11 for *Thada*-SAM). (**g**) The $[Ca^{2+}]_i$ in dispersed islet cells from WT and *Thada*-KO mice before and after 10 μM IP3 stimulation under 11.1 mM glucose (n=30 from six mice each group). (**h**) Quantification of the maximal increase in $[Ca^{2+}]_i$ after IP3 stimulation (n=30 from six mice each group). (**i**) Western blot analysis of THADA protein expression in control and six subclones of *Thada*-KO MIN6 beta-cell lines. (**j** and **k**) Quantification of the maximum increases in $[Ca^{2+}]_i$ from control (n=14) and *Thada*-KO MIN6 beta-cell lines (n=13) after 5 μM ionomycin stimulation. Representative results of two replicates from each group are provided. (**l** and **m**) Quantification of the maximum increases in $[Ca^{2+}]_i$ from control (n=19) and *Thada*-KO MIN6 beta-cell lines (n=18) after 1 μM thapsigargin stimulation. Representative results of two replicates from each group are provided. Data are presented as mean ± SEM. **P*<0.05, ***P*<0.01, ****P*<0.001; two-tailed unpaired Student's *t* test. Source data are provided as a source data file.

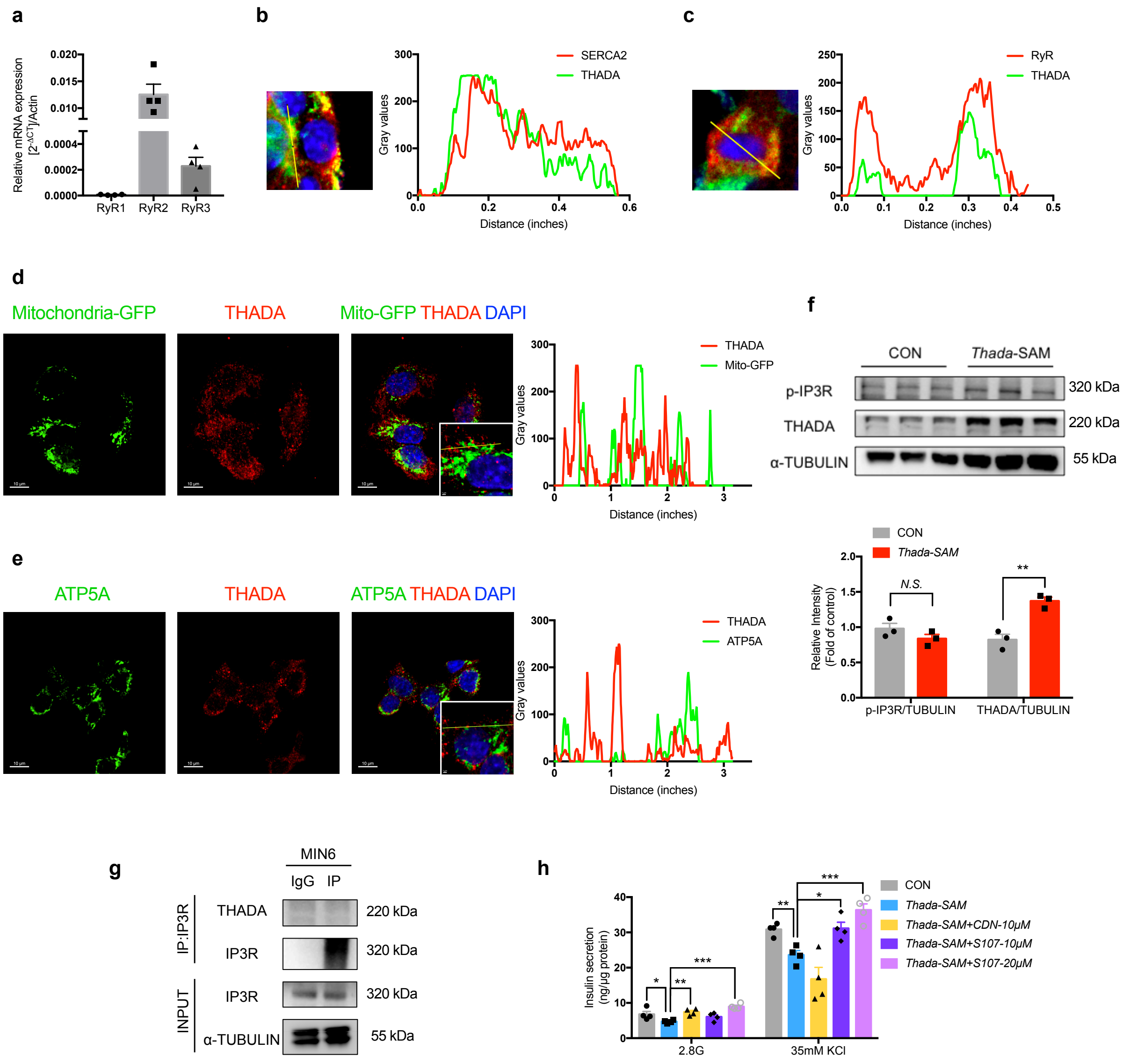

Figure S6. THADA impairs insulin secretion through SERCA2 and RyR2 in β-cells.

(**a**) Detection of the mRNA expression levels of *RyR1*, *RyR2* and *RyR3* in MIN6 beta-cell line (n=4). (**b**) Co-localization analysis of immunofluorescence stained for THADA (green) and SERCA2 (red) in Fig. 5m. (**c**) Co-localization analysis of immunofluorescence stained for THADA (green) and RyR (red) in Fig. 5n. (**d**) Immunofluorescence staining and co-localization analysis for Mitochondria-GFP (green), THADA (red), and DAPI (blue) in MIN6 beta-cell line. Scale bars, 10 μm. (**e**) Immunofluorescence staining and co-localization analysis for ATP5A (green), THADA (red), and DAPI (blue) in MIN6 beta-cell line. Scale

bars, 10 μm. (**f**) Western blot analysis and quantification of phosphorylated IP3R protein levels in control and *Thada*-SAM MIN6 beta-cell lines (n=3). (**g**) MIN6 cells were immunoprecipitated with either a IP3R antibody or an IgG negative control, followed by western blot analysis with a THADA antibody. (**h**) Control and *Thada*-SAM MIN6 beta-cell lines were pretreated with or without CDN1163 and S107 at the indicated concentration for 24 h, then stimulated with or without 35 mM KCl at 2.8 mM glucose for 1 h, and insulin secretion was assayed (n=4). All western blots and immunostainings show representative results from at least three independent experiments. Data are presented as mean ± SEM. $^{*}P<0.05$, $^{**}P<0.01$, $^{***}P<0.001$; two-tailed unpaired Student's *t* test. Source data are provided as a source data file.

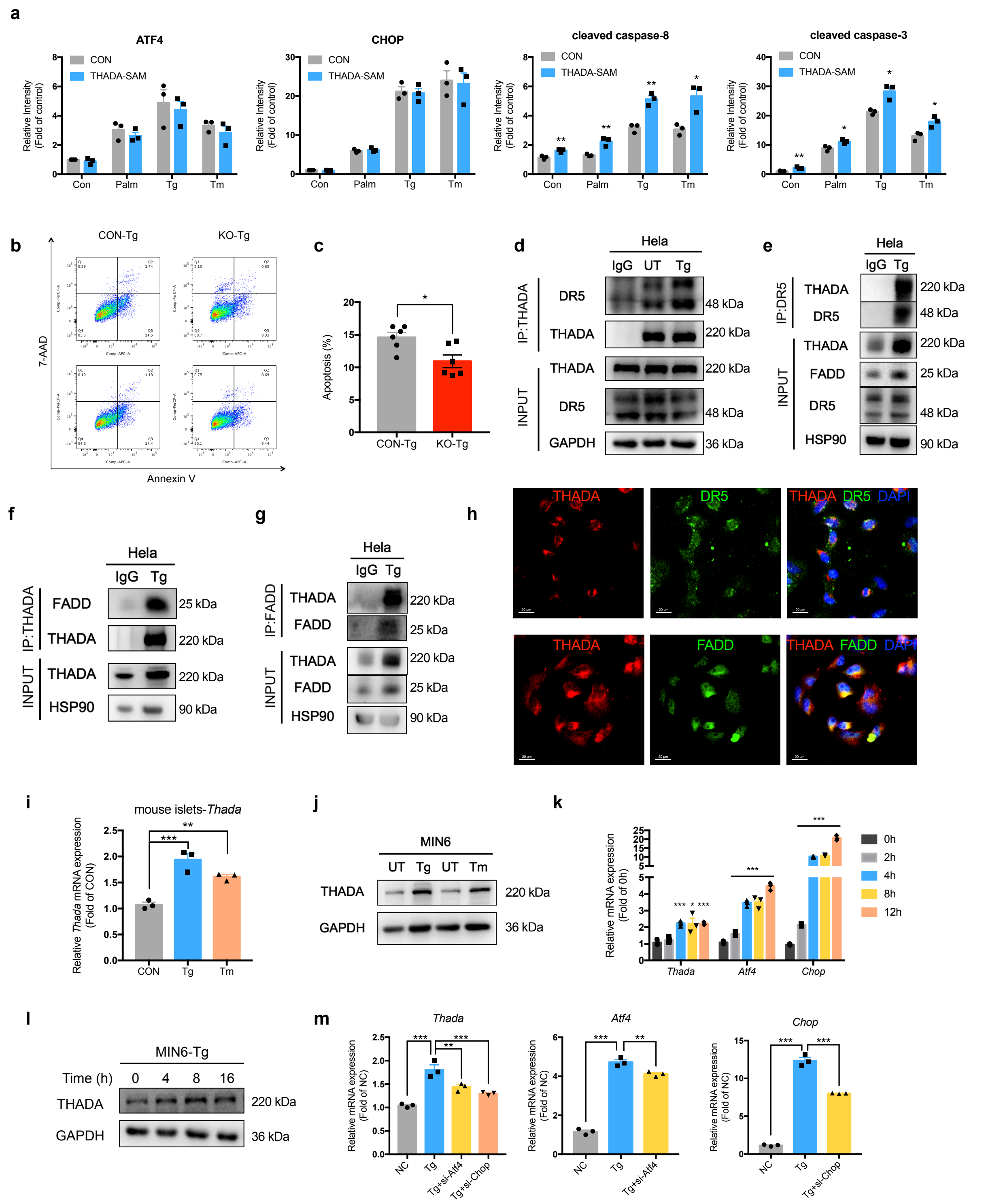

Figure S7. THADA aggravates ER stress-induced apoptosis by activating DR5/FADD/caspase-8.

(**a**) Control and *Thada*-activated β-cells were treated with or without 0.5 mM palmitate (Palm), 1 μM thapsigargin (Tg) or 1 μg/ml tunicamycin (Tm) for 24 h, then protein intensities

of cleaved caspase-8, cleaved caspase-3, CHOP and ATF4 were determined (n=3 independent experiments). (**b**) Flow cytometry analysis of control and *Thada*-KO MIN6 beta-cell lines treated with 1 μM Tg for 24 h. Representative dot plots of cell apoptosis were shown after dual staining with Annexin V and 7-AAD. (**c**) The apoptosis of control and *Thada*-KO MIN6 beta-cell lines after Tg treatment were quantified by the percentage of Annexin V-positive cells (n=6). (**d**) HeLa cells were untreated (UT) or treated with 1 μM Tg for 24 h, then immunoprecipitated with either an IgG control or THADA antibody, followed by western blot analysis with DR5 antibodies. (**e**) HeLa cells were treated with 1 μM Tg for 24 h, then immunoprecipitated with either an IgG control or DR5 antibody, followed by western blot analysis with THADA antibodies. (**f**) HeLa cells were treated with 1 μM Tg for 24 h, then immunoprecipitated with either an IgG control or THADA antibody, followed by western blot analysis with FADD antibodies. (**g**) HeLa cells were treated with 1 μM Tg for 24 h, then immunoprecipitated with either an IgG control or FADD antibody, followed by western blot analysis with THADA antibodies. (**h**) HeLa cells were treated with 1 μM Tg for 24 h, then immunofluorescence was performed by staining THADA (red) and DR5 (green), or THADA (red) and FADD (green). Scale bars, 20 μm. (**i**) Mouse islets were treated with 1 μM Tg or 1 μg/ml Tm for 24 h, then *Thada* mRNA expression was determined. (**j**) THADA protein level was determined after MIN6 beta-cell lines were treated with 1 μM Tg or 1 μg/ml Tm for 24 h. (**k**) *Thada*, *Atf4*, and *Chop* mRNA levels were determined after MIN6 beta-cell lines were treated with 1 μM Tg for 0, 2, 4, 8, or 12h (n=3). (**l**) THADA protein level was determined after MIN6 beta-cell lines were treated with 1 μM Tg for 0, 4, 8, or 16 h. (**m**) *Thada*, *Atf4*, and *Chop* mRNA levels were determined after MIN6 beta-cell lines were transfected with siRNA

targeting *Atf4* or *Chop*, and then treated with 1 µM Tg for 24h (n=4 for *Thada*, n=3 for *Atf4* and *Chop*). All western blots and immunostainings show representative results from at least three independent experiments. Data are presented as mean ± SEM. **P*<0.05, ***P*<0.01, ****P*<0.001; two-tailed unpaired Student's *t* test (a, c, k) or one-way ANOVA followed by Dunnett's multiple test (i, m). Source data are provided as a source data file.

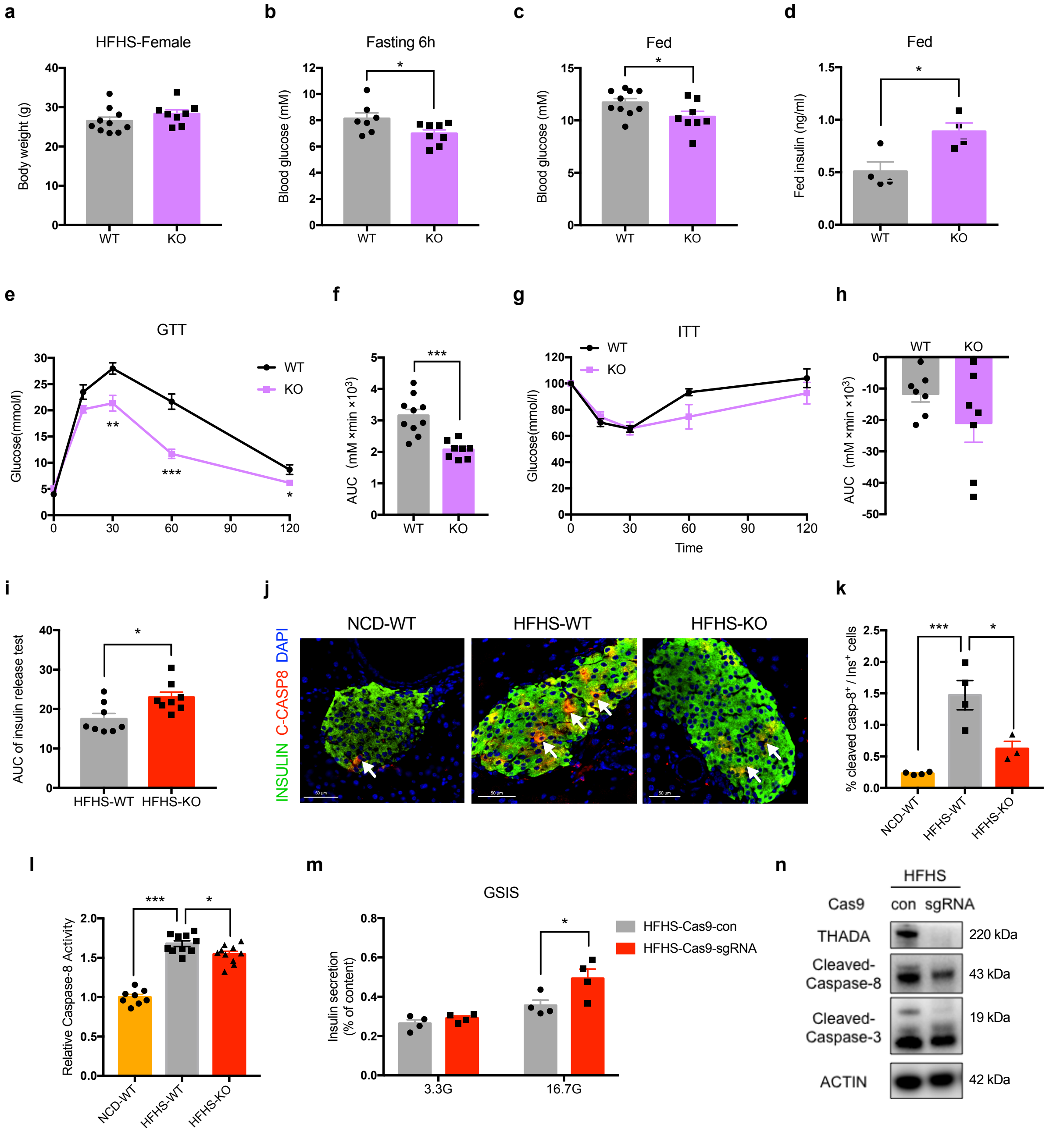

Figure S8. *Thada* knockout protects mice from HFHS diet-induced glucose intolerance. (**a**) Body weights of WT (n=10) and *Thada*-KO female mice (n=8) after HFHS diet for 12 weeks. (**b**) Blood glucose levels of HFHS-fed WT (n=7) and *Thada*-KO female mice that were fasted for 6 h (n=8). (**c**) Fed blood glucose levels of HFHS-fed WT (n=10) and *Thada*-KO female mice (n=8). (**d**) Fed serum insulin levels of HFHS-fed WT and *Thada*-KO female mice (n=4). (**e** and **f**) IPGTT and area under the curve of HFHS-fed WT (n=10) and *Thada*-KO female mice (n=8). (**g** and **h**) ITT and area under baseline of HFHS-fed WT and *Thada*-KO female mice (n=7). (**i**) AUC of the insulin release test of HFHS-fed mice in Fig. 7i (n=8 mice). (**j**) Representative immunofluorescence images of islets from normal chow diet (NCD)-fed WT, HFHS-fed WT, and HFHS-fed *Thada*-KO male mice stained for insulin (green), cleaved caspase-8 (red), and DAPI (blue). Scale bars, 50 μm. Arrowhead points to cleaved caspase-8^+Insulin$^+$ cells. (**k**) The percentage of cleaved caspase-8^+ in Insulin$^+$ cells was quantified (n=4 mice for NCD-WT and HFHS-WT, n=3 mice for HFHS-KO). At least 2500 Insulin$^+$ cells in each mouse were counted for quantification. (**l**) The caspase-8 activities of islets from NCD-fed WT (n=8), HFHS-fed WT (n=10), and HFHS-fed *Thada*-KO (n=10) male mice were assayed. (**m** and **n**) Islets were isolated from male mice after HFHS feeding for 20 weeks and infected with lentivirus encoding Cas9-control or Cas9-sgRNA targeting *Thada*. Then GSIS (m, n=4), cleaved caspase-8 and caspase-3 protein levels were determined (n). The experiments were performed on mice after HFHS diet for 12 weeks unless otherwise indicated. Data are presented as mean ± SEM. **P*<0.05, ***P*<0.01, ****P*<0.001; two-tailed unpaired Student's *t* test (a-i, m) or one-way ANOVA followed by Dunnett's multiple test (k, l). Source data are provided as a source data file.

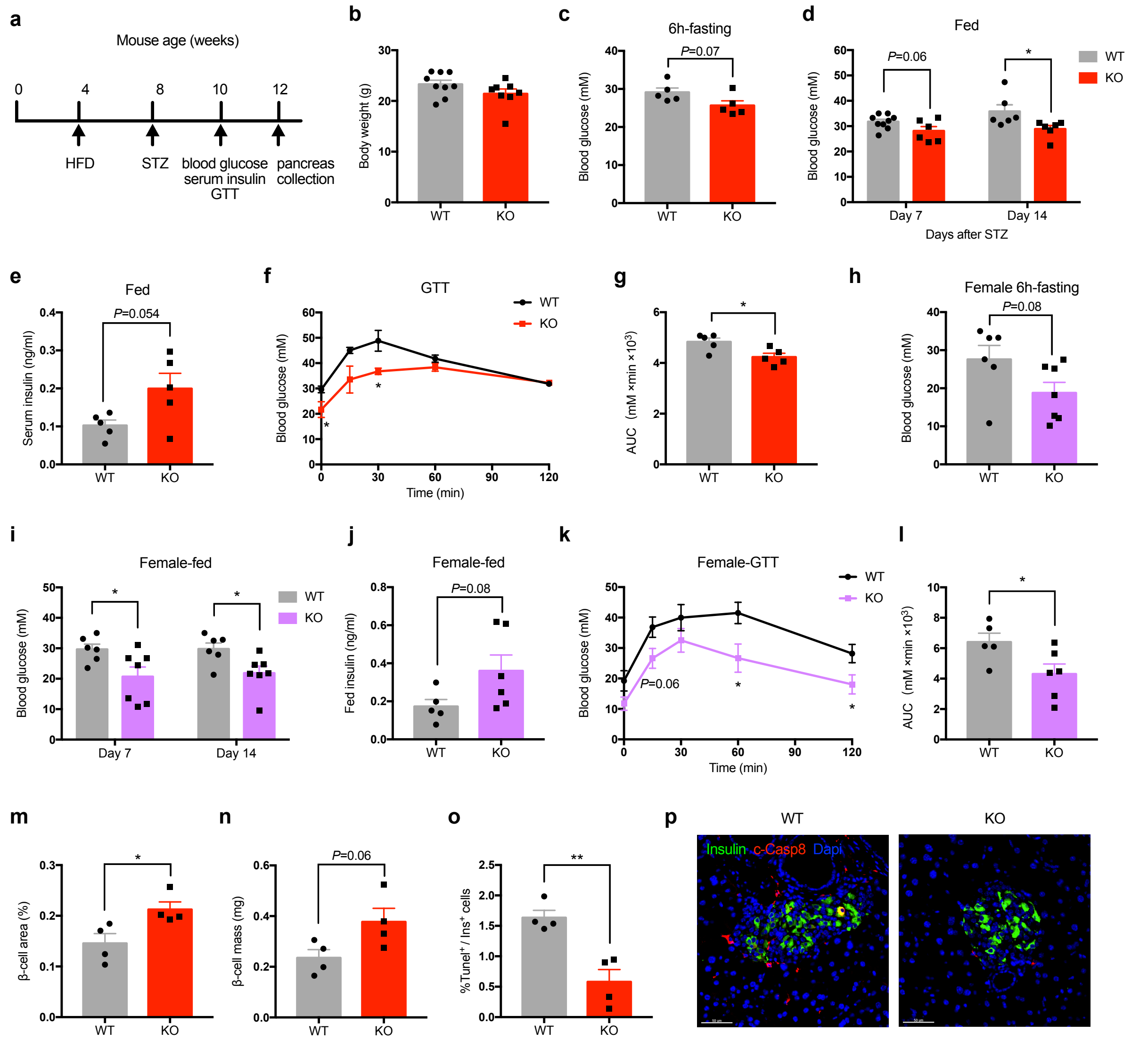

Figure S9. *Thada* knockout protects mice from HFD/STZ-induced hyperglycemia and β-cell loss.

(**a**) Schematic design of the HFD/STZ mouse model studies. 4-week-old WT and *Thada*-KO mice were fed with an HFD diet. After 4 weeks, mice were injected with a single-dose of STZ (100mg/kg) and then metabolic phenotypes were analyzed. (**b**) Body weights of WT (n=9) and *Thada*-KO male mice (n=8) after STZ injection for 14 days. (**c**) Blood glucose levels of 6-h fasted WT and *Thada*-KO male mice after STZ injection for 14 days (n=5). (**d**) Fed blood glucose levels of WT and *Thada*-KO male mice after STZ injection for 7 and 14 days (n=9

for WT at 7 days, n=6 for WT at 14 days, n=6 for KO). (**e**) Fed serum insulin levels of WT and *Thada*-KO male mice after STZ injection for 14 days (n=5). (**f** and **g**) IPGTT and area under the curve of WT and *Thada*-KO male mice after STZ injection for 14 days (n=5). (**h**) Blood glucose levels of 6-h fasted WT (n=6) and *Thada*-KO (n=7) female mice after STZ injection for one week. (**i**) Fed blood glucose levels of WT (n=6) and *Thada*-KO (n=7) female mice after STZ injection for 7 and 14 days. (**j**) Fed serum insulin levels of WT (n=5) and *Thada*-KO (n=6) female mice after STZ injection for 14 days. (**k** and **l**) IPGTT and area under the curve of WT (n=5) and *Thada*-KO (n=6) female mice after STZ injection for 14 days. (**m**) Measurements of β-cell area/pancreatic area ratio from WT and *Thada*-KO male mice after STZ injection for 4 weeks (n=4). (**n**) Measurements of β-cell mass from WT and *Thada*-KO male mice after STZ injection for 4 weeks (n=4). (**o**) Quantification of Tunel-positive β-cells from WT and *Thada*-KO male mice after STZ injection for 4 weeks (n=4). (**p**) Representative immunofluorescence images of islets from WT and *Thada*-KO male mice after STZ injection for 4 weeks stained for insulin (green), cleaved caspase-8 (red) and DAPI (blue). Data are presented as mean ± SEM. *P<0.05, **P<0.01; two-tailed unpaired Student's t test. Source data are provided as a source data file.

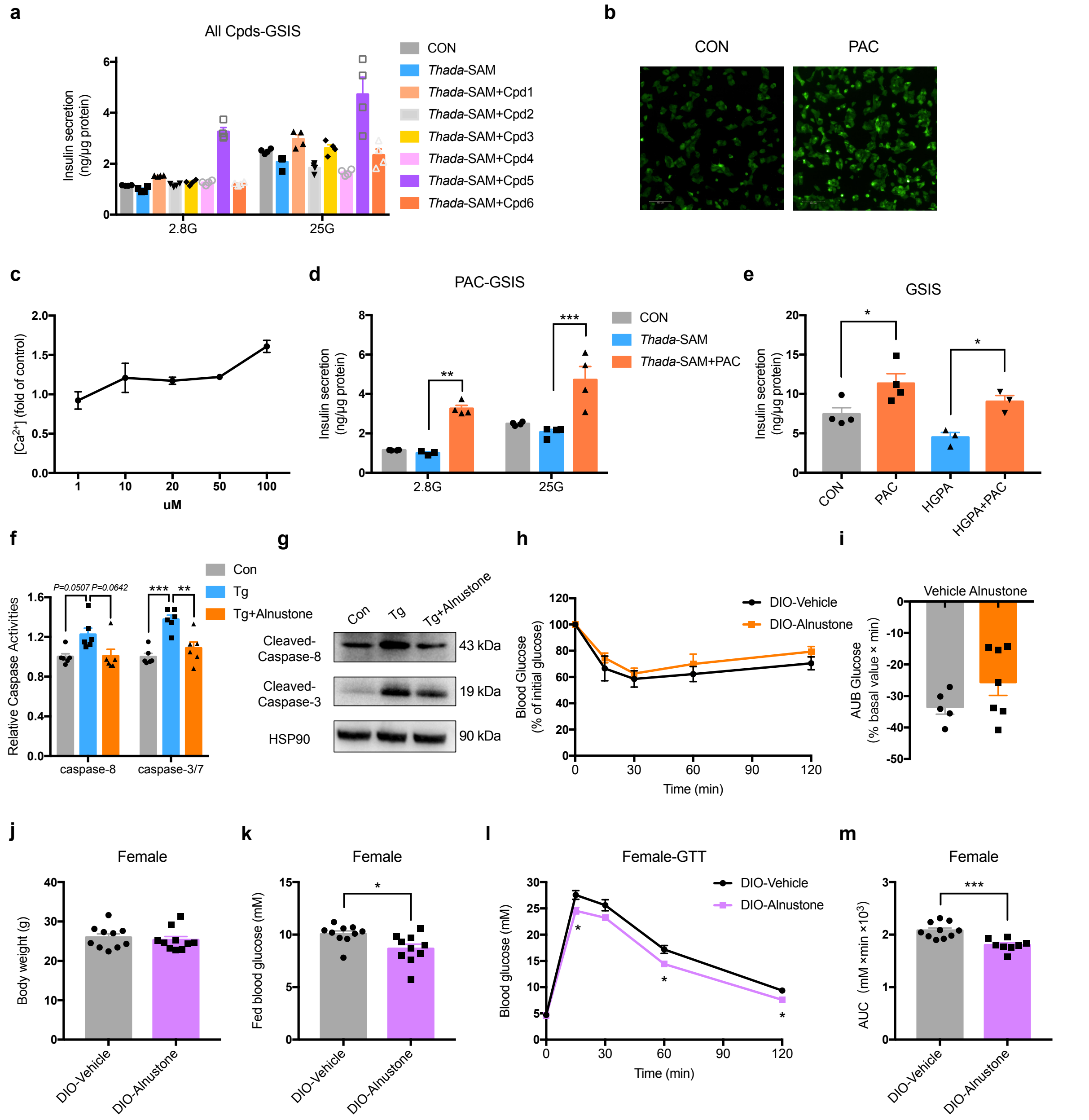

Figure S10. A high-content screen identifies proanthocyanidin and alnustone that can reverse THADA-induced β-cell dysfunction.

(**a**) *Thada*-SAM β-cells were treated with or without the indicated top six compounds for 24 h, then GSIS was assayed. Compound 1 is alnustone and compound 5 is proanthocyanidin. (**b**) Representative images of glucose-stimulated fluo-4 Ca^{2+} fluorescence after treatment with vehicle or proanthocyanidin (PAC) for 24 h. Scale bar, 100 μm. (**c**) Dose curve of proanthocyanidin on the glucose-stimulated $[Ca^{2+}]_I$ (n=3 biological replicates). (**d**) GSIS of

Thada-SAM β-cells treated with or without 10 μM proanthocyanidin for 24h (n=4 biologically independent experiments). (**e**) MIN6 beta-cell lines were treated with or without 33 mM glucose and 0.5 mM palmitate (HGPA) in the presence or absence of 10 μM proanthocyanidin for 24h. Then GSIS was assayed (n=4 biologically independent experiments). (**f**) The caspase-8 and caspase-3/7 activities of MIN6 beta-cell lines were assayed after treatment with 1 μM Tg in the presence or absence of 10 μM alnustone for 24 h (n=6). (**g**) MIN6 beta-cell lines were treated with 1 μM Tg in the presence or absence of 10 μM alnustone for 24 h, then cleaved caspase-8 and cleaved caspase-3 protein levels were determined. Data shows representative result from at least three independent experiments. (**h** and **i**) DIO male mice were injected with vehicle or 10 mg/kg alnustone for 7 days, then ITT and the related AUB were measured (n=5 for vehicle and n=7 for alnustone). (**j**-**m**) DIO female mice were injected with vehicle or 10 mg/kg alnustone for 5-7 days, then body weights (j, n=10), fed blood glucose levels (k, n=10), IPGTT and the related AUC (l and m, n=10 for vehicle and n=8 for alnustone) were measured. Data are presented as mean ± SEM. **P*<0.05, ***P*<0.01, ****P*<0.001; two-way ANOVA followed by Tukey's multiple comparison test (d, f) or two-tailed unpaired Student's *t* test (e, h-m). Source data are provided as a source data file.
