# Supplementary material for: THADA inhibition in mice protects against type 2 diabetes mellitus by improving pancreatic β-cell function and preserving β-cell mass
Source: Nat Commun. 2023 Feb 23;14:1020. doi: 10.1038/s41467-023-36680-0 (PMC9950491; doi:10.1038/s41467-023-36680-0)
Supplement: Supplementary file 2 — Description of Additional Supplementary Files [file 41467_2023_36680_MOESM2_ESM.pdf]

Description of Additional Supplementary Files

File Name: Supplementary Data 1
Description: clinical characteristics of human participants.

File Name: Supplementary Data 2
Description: Sequences of the primers.
