# Supplementary material for: THADA inhibition in mice protects against type 2 diabetes mellitus by improving pancreatic β-cell function and preserving β-cell mass
Source: Nat Commun. 2023 Feb 23;14:1020. doi: 10.1038/s41467-023-36680-0 (PMC9950491; doi:10.1038/s41467-023-36680-0)
Supplement: Supplementary file 5 — Reporting Summary [file 41467_2023_36680_MOESM5_ESM.pdf]

nature portfolio

Corresponding author(s): Han Zhao

Last updated by author(s): Jan 16, 2023

Reporting Summary

Nature Portfolio wishes to improve the reproducibility of the work that we publish. This form provides structure for consistency and transparency in reporting. For further information on Nature Portfolio policies, see our Editorial Policies and the Editorial Policy Checklist.

Statistics

For all statistical analyses, confirm that the following items are present in the figure legend, table legend, main text, or Methods section.

n/a	Confirmed	
☐	☒	The exact sample size (*n*) for each experimental group/condition, given as a discrete number and unit of measurement
☐	☒	A statement on whether measurements were taken from distinct samples or whether the same sample was measured repeatedly
☐	☒	The statistical test(s) used AND whether they are one- or two-sided<br>*Only common tests should be described solely by name; describe more complex techniques in the Methods section.*
☒	☐	A description of all covariates tested
☐	☒	A description of any assumptions or corrections, such as tests of normality and adjustment for multiple comparisons
☐	☒	A full description of the statistical parameters including central tendency (e.g. means) or other basic estimates (e.g. regression coefficient) AND variation (e.g. standard deviation) or associated estimates of uncertainty (e.g. confidence intervals)
☐	☒	For null hypothesis testing, the test statistic (e.g. *F*, *t*, *r*) with confidence intervals, effect sizes, degrees of freedom and *P* value noted<br>*Give P values as exact values whenever suitable.*
☒	☐	For Bayesian analysis, information on the choice of priors and Markov chain Monte Carlo settings
☒	☐	For hierarchical and complex designs, identification of the appropriate level for tests and full reporting of outcomes
☒	☐	Estimates of effect sizes (e.g. Cohen's *d*, Pearson's *r*), indicating how they were calculated

Our web collection on statistics for biologists contains articles on many of the points above.

Software and code

Policy information about availability of computer code

Data collection	Harmony 4.9 software (PerkinElmer) was used to collect the high-content screening data. Fluo-4 calcium fluorescence was measured using an Enspire system (PerkinElmer).
Data analysis	Statistical analyses were performed using GraphPad Prism 7 software (GraphPad Software, Inc.). Western blot image quantifications were performed using ImageJ software (NIH). Flow cytometric analyses were performed with FlowJo v10 software (BD Biosciences). Fura-2 calcium images were analysed using MetaFluor software (Molecular Devices). High-content screening images were analysed using Harmony 4.9 software (PerkinElmer).

For manuscripts utilizing custom algorithms or software that are central to the research but not yet described in published literature, software must be made available to editors and reviewers. We strongly encourage code deposition in a community repository (e.g. GitHub). See the Nature Portfolio guidelines for submitting code & software for further information.

Data

Policy information about availability of data

All manuscripts must include a data availability statement. This statement should provide the following information, where applicable:

- Accession codes, unique identifiers, or web links for publicly available datasets
- A description of any restrictions on data availability
- For clinical datasets or third party data, please ensure that the statement adheres to our policy

All data generated for this study are available from the corresponding author upon reasonable request. RNA sequencing data generated in this study have been deposited in the Gene Expression Omnibus database under accession code GSE173267 (https://www.ncbi.nlm.nih.gov/geo/query/acc.cgi?acc=GSE173267).

Human research participants

Policy information about studies involving human research participants and Sex and Gender in Research.

Reporting on sex and gender	Both male and female sexes were considered in study design and included in the analyses. The disaggregated sex and gender data have been provided in the supplementary table, and consent from eight individuals have been obtained for sharing of individual-level data.
Population characteristics	The characteristics for each human participants were described in detail in Supplementary table 1.
Recruitment	This study did not involve recruitment.
Ethics oversight	All human studies and protocols used were approved by the Biomedical Research Ethic Committee of Shandong Provincial Hospital.

Note that full information on the approval of the study protocol must also be provided in the manuscript.

Field-specific reporting

Please select the one below that is the best fit for your research. If you are not sure, read the appropriate sections before making your selection.

☒ Life sciences ☐ Behavioural & social sciences ☐ Ecological, evolutionary & environmental sciences

For a reference copy of the document with all sections, see nature.com/documents/nr-reporting-summary-flat.pdf

Life sciences study design

All studies must disclose on these points even when the disclosure is negative.

Sample size	No statistical method was used to predetermine animal's sample size. The sample size was determined based on experience with the used experimental models/setups.
Data exclusions	No data were excluded from the analyses.
Replication	Replication attempts were successful. For in vivo experiments,at least 3 animals per group were used (exact numbers are reported in image legends). All in vitro experiments were performed in at least 3 independent experiments.
Randomization	Animals were randomly assigned to experimental groups and matched for age and gender.
Blinding	The metabolic phenotype tests of mice were performed blindly.

Reporting for specific materials, systems and methods

We require information from authors about some types of materials, experimental systems and methods used in many studies. Here, indicate whether each material, system or method listed is relevant to your study. If you are not sure if a list item applies to your research, read the appropriate section before selecting a response.

Materials & experimental systems

n/a	Involved in the study
☐	☒ Antibodies
☐	☒ Eukaryotic cell lines
☒	☐ Palaeontology and archaeology
☐	☒ Animals and other organisms
☒	☐ Clinical data
☒	☐ Dual use research of concern

Methods

n/a	Involved in the study
☒	☐ ChIP-seq
☐	☒ Flow cytometry
☒	☐ MRI-based neuroimaging

Antibodies

Antibodies used

Antibodies used for western-blotting (all 1:1000 dilution):
anti-THADA (Sigma #HPA035192), anti-SERCA2 (Abcam #ab2861), anti-RyR (Invitrogen, #MA3-916), anti-cleaved caspase-8 (Cell Signaling Technology #8592), anti-caspase-8 (Cell Signaling Technology #4927), anti-cleaved caspase-3 (Cell Signaling Technology #9664), anti-ATF4 (Cell Signaling Technology #11815), anti-CHOP (Cell Signaling Technology #2895), anti-FADD (Millipore #05-486), anti-DR5 (Abcam, #ab8416), anti-phospho-IP3R (Cell Signaling Technology #3760), anti-IP3R (Santa Cruz #sc377518), anti-HSP90 (Cell Signaling Technology #4874), anti-GAPDH (Proteintech #60004-1-Ig), anti-β-Actin (Proteintech #60008-1-Ig).
Antibodies used for immunostaning:
guinea pig anti-insulin (DAKO, #IR00261-2), rabbit anti-THADA (Sigma, #HPA035192), mouse anti-glucagon (Abcam, #ab10988), rabbit anti-Ki67 (Cell Signaling Technology, #9129), mouse anti-SERCA2 (Abcam, #ab2861), mouse anti-RyR (Invitrogen, #MA3-916), mouse anti-DR5 (Santa Cruz, SC-166624), mouse anti-FADD (Millipore, #05-486), mouse anti-ATP5A (Abcam, #ab14748).

Validation

Antibody validation information was available from the manufacturers.
anti-THADA (https://www.sigmaaldrich.cn/CN/zh/product/sigma/hpa035192?context=product),
anti-RyR (https://www.thermofisher.cn/cn/zh/antibody/product/Ryanodine-Receptor-Antibody-clone-C3-33-Monoclonal/MA3-916)
anti-cleaved caspase-8 (https://www.cellsignal.cn/products/primary-antibodies/cleaved-caspase-8-asp387-d5b2-xp-rabbit-mab-mouse-specific/8592?site-search-type=Products&N=4294956287&Ntt=8592&fromPage=plp&_requestid=27518),
anti-caspase-8 (https://www.cellsignal.com/products/primary-antibodies/caspase-8-antibody-mouse-specific/4927),
anti-cleaved caspase-3 (https://www.cellsignal.com/products/primary-antibodies/cleaved-caspase-3-asp175-5a1e-rabbit-mab/9664),
anti-ATF4 (https://www.cellsignal.com/products/primary-antibodies/atf-4-d4b8-rabbit-mab/11815),
anti-CHOP (https://www.cellsignal.com/products/primary-antibodies/chop-l63f7-mouse-mab/2895),
anti-FADD (https://www.merckmillipore.com/CN/zh/product/Anti-FADD-Antibody-clone-1F7,MM_NF-05-486),
anti-DR5 (https://www.abcam.cn/dr5-antibody-ab8416.html),
anti-phospho-IP3R(https://www.cellsignal.cn/products/primary-antibodies/phospho-ip3-receptor-ser1756-antibody/3760?site-search-type=Products&N=4294956287&Ntt=3760&fromPage=plp&_requestid=426983)
anti-IP3R (https://www.scbt.com/zh/p/ip3r-i-ii-iii-antibody-b-2
anti-HSP90 (https://www.cellsignal.com/products/primary-antibodies/hsp90-antibody/4874),
anti-GAPDH (http://www.ptgcn.com/products/GAPDH-Antibody-60004-1-Ig.htm),
anti-β-Actin (http://www.ptgcn.com/products/ACTB-Antibody-60008-1-Ig.htm),
rabbit anti-Ki67 (https://www.cellsignal.com/products/primary-antibodies/ki-67-d3b5-rabbit-mab/9129),
anti-ATP5A (https://www.abcam.cn/atp5a-antibody-15h4c4-mitochondrial-marker-ab14748.html).

Eukaryotic cell lines

Policy information about cell lines and Sex and Gender in Research

Cell line source(s)

MIN6 and INS-1 cells were generous gifts from Prof. Xiao Wang (Ruijin hospital, Shanghai Jiao Tong University School of Medicine). HeLa cell was purchased from National Collection of Authenticated Cell Cultures (Catalog: TCHu187) that was derived from a female cervical adenocarcinoma.

Authentication

MIN6 and INS-1 cell lines were authenticated in-lab by performing glucose-stimulated insulin secretion and RT-PCR of beta-cell specific genes.

Mycoplasma contamination

All cell lines were tested negative for mycoplasma.

Commonly misidentified lines
(See ICLAC register)

No commonly misidentified cell lines were used.

Animals and other research organisms

Policy information about studies involving animals; ARRIVE guidelines recommended for reporting animal research, and Sex and Gender in Research

Laboratory animals

C57BL/6J mice were purchased from Vital River Laboratories (Beijing, China). Both male and female mice were used at the indicated ages. Global Thada-knockout mice, Thada-floxed mice and Ins1-Cre-Dsred mice were all maintained on C57BL/6 background, with the sex and age described in the figure legend.

Wild animals

No wild animals are included in this study.

Reporting on sex	Both male and female sexes were considered in study design and included in the animal experiments. The sex and number of the animals have been described in the figure legend, and the indicated data have been provided in the source data file.
Field-collected samples	Our study did not involve field-collections.
Ethics oversight	All animal experiments were performed in accordance with the approval of the Animal Ethics Committee of the School of Medicine, Shandong University (SDULCLL2021-2-18).

Note that full information on the approval of the study protocol must also be provided in the manuscript.

Flow Cytometry

Plots

Confirm that:

- [x] The axis labels state the marker and fluorochrome used (e.g. CD4-FITC).
- [x] The axis scales are clearly visible. Include numbers along axes only for bottom left plot of group (a 'group' is an analysis of identical markers).
- [x] All plots are contour plots with outliers or pseudocolor plots.
- [x] A numerical value for number of cells or percentage (with statistics) is provided.

Methodology

Sample preparation	For cell apoptosis analyses, cells were trypsinized, washed with PBS, resuspended in binding buffer and then incubated with Annexin V-APC and 7-AAD for ten minutes following the manufacturer's instructions.
Instrument	LSR Fortessa flow cytometer (BD Biosciences)
Software	FlowJo v10
Cell population abundance	10,000 events were recorded for cell apoptosis analyses.
Gating strategy	Starting cell population was determined by FSC-A/SSC-A gating. Singlets were gated according to the pattern of FSC-A/FSC-H. Positive/Negative populations were determined by untreated controls.

- [x] Tick this box to confirm that a figure exemplifying the gating strategy is provided in the Supplementary Information.
